# A metabolic associated fatty liver disease risk variant in MBOAT7 regulates toll like receptor induced outcomes

**DOI:** 10.1038/s41467-022-35158-9

**Published:** 2022-12-06

**Authors:** Jawaher Alharthi, Ali Bayoumi, Khaled Thabet, Ziyan Pan, Brian S. Gloss, Olivier Latchoumanin, Mischa Lundberg, Natalie A. Twine, Duncan McLeod, Shafi Alenizi, Leon A. Adams, Martin Weltman, Thomas Berg, Christopher Liddle, Jacob George, Mohammed Eslam

**Affiliations:** 1 Storr Liver Centre, Westmead Institute for Medical Research, Westmead Hospital and University of Sydney, Sydney, NSW Australia; 2 Department of Biotechnology, Faculty of Science, Taif University, Taif, Saudi Arabia; 3 Department of Biochemistry, Faculty of Pharmacy, Minia University, Minia, 6111 Egypt; 4 Westmead Research Hub, Westmead Institute for Medical Research, Sydney, NSW Australia; 5 Transformational Bioinformatics, Commonwealth Scientific and Industrial Research Organisation, Sydney, NSW Australia; 6 The University of Queensland Diamantina Institute, The University of Queensland, Woolloongabba, QLD 4102 Australia; 7 The University of Queensland Faculty of Medicine, Brisbane, QLD Australia; 8 Department of Anatomical Pathology, Institute of Clinical Pathology and Medical Research (ICPMR), Westmead Hospital, Sydney, NSW Australia; 9 Medical School, Sir Charles Gairdner Hospital Unit, University of Western Australia, Nedlands, WA Australia; 10 Department of Gastroenterology and Hepatology, Nepean Hospital, Sydney, NSW Australia; 11 Division of Hepatology, Department of Medicine II, Leipzig University Medical Center, Leipzig, Germany

**Keywords:** Non-alcoholic fatty liver disease, Translational research

## Abstract

The breakdown of toll-like receptor (TLR) tolerance results in tissue damage, and hyperactivation of the TLRs and subsequent inflammatory consequences have been implicated as risk factors for more severe forms of disease and poor outcomes from various diseases including COVID-19 and metabolic (dysfunction) associated fatty liver disease (MAFLD). Here we provide evidence that membrane bound O-acyltransferase domain containing 7 (MBOAT7) is a negative regulator of TLR signalling. MBOAT7 deficiency in macrophages as observed in patients with MAFLD and in COVID-19, alters membrane phospholipid composition. We demonstrate that this is associated with a redistribution of arachidonic acid toward proinflammatory eicosanoids, induction of endoplasmic reticulum stress, mitochondrial dysfunction, and remodelling of the accessible inflammatory-related chromatin landscape culminating in macrophage inflammatory responses to TLRs. Activation of MBOAT7 reverses these effects. These outcomes are further modulated by the *MBOAT7* rs8736 (T) MAFLD risk variant. Our findings suggest that MBOAT7 can potentially be explored as a therapeutic target for diseases associated with dysregulation of the TLR signalling cascade.

## Abstract (web summary)

Hyperactivation of the toll-like receptors (TLRs) have been implicated as risk factors for more severe forms of disease in COVID-19 and metabolic (dysfunction) associated fatty liver disease (MAFLD). Here the authors report that MBOAT7 is reduced in macrophages of patients with MAFLD and COVID-19, and acts as a negative regulator of TLR signalling.

## Introduction

Macrophages represent a crucial cell type for initial host responses to pathogens given their high expression of pattern recognition receptors (PRRs) such as Toll-like receptors (TLRs)^1^. Although required for effective immunity, overactivation of TLR signalling or breakdown of TLR tolerance produces a large quantity of inflammatory cytokines and chemokines which will provoke inflammation and ultimately results in tissue damage. Thus, tight regulation of TLRs signalling is critical to avoid unchecked amplification of inflammation and for maintaining tissue homoeostasis^2–4^.

Hyperactivation of the TLRs and subsequent inflammatory consequences have been implicated as risk factors for more severe forms of disease and poor outcomes from various disease including COVID-19 and metabolic (dysfunction) associated fatty liver disease (MAFLD)^5–7^. Identification of regulators of TLRs signalling is pivotal and can pave the path for novel therapeutic drug development.

A variant in the Membrane Bound O-Acyltransferase Domain Containing 7 (*MBOAT7)* gene that is abundantly expressed in inflammatory cells has been identified by genome-wide association and candidate gene studies in inflammation and liver injury across multiple liver diseases including MAFLD, alcoholic fatty liver disease, and viral hepatitis^8–11^. *MOBAT7* is also implicated in COVID-19 severity^12^.

MBOAT7 is anchored to endomembranes by six transmembrane (TM) domains^13^ and encodes lysophosphatidylinositol acyltransferase 1 (LPIAT1) which preferentially incorporates arachidonic acid into phosphatidylinositol (PI)^14^. PI is a constituent of membrane phospholipids that are critical for the switch that initiates stress responses^15,16^. Of relevance, TLR stimulation alters macrophage lipid homoeostasis^17^ enhancing mitochondrial reactive oxygen species (ROS) generation^18^. Though, MBOAT7 is expressed in immune cells, the role of MBOAT7 in these cells and how the risk variant functionally contributes to disease severity is unknown. We questioned if MBOAT7 is a novel regulator of TLRs-initiated outcomes, and if so, the underlying mechanism(s). We also reasoned that these outcomes would be modulated by MBOAT7 genotype. If proven, the findings would help link metabolic dysregulation in MAFLD to unbalanced immune responses, and to severe COVID-19.

Consistent with our hypotheses, here we show that MBOAT7 is an essential negative regulator of Toll-like receptor signalling. MBOAT7 expression is reduced in both patients with MAFLD and COVID-19 and in human macrophages after TLRs and SARS-CoV-2 stimulation. This decrease in expression levels aggravates TLR and SARS-CoV-2 induced inflammatory responses, while overexpression of MBOAT7 reverses these effects. Functionally, MBOAT7 deficiency in macrophages alters membrane phospholipid (PL) composition thereby aggravating endoplasmic reticulum stress and intracellular calcium resulting in alterations of mitochondrial function. Reduced incorporation of arachidonic acid (AA) into PLs was associated with a redistribution of arachidonic acid toward proinflammatory eicosanoids. Finally, we show that depletion of MBOAT7 causes a rewiring in the inflammation-associated chromatin landscape that primes cells for augmented proinflammatory cytokine production. Our analysis of macrophages and liver samples from patients with MAFLD as well as Phenome-wide association studies (PheWAS) corroborate these observations.

## Results

### MBOAT7 is downregulated in human MDMs and Kupffer cells upon TLRs stimulation

TLRs play a crucial role in MAFLD pathogenesis through activation of several inflammation and cytokine-related pathways^19,20^. Given the high expression of MBOAT7 in immune cells^9^, we explored the effect of TLR signalling modulation on MBOAT7 expression. As TLR4 signalling is an important mediator of inflammation in MAFLD^19,20^, we first examined the relationship between this TLR and MBOAT7. In MDMs, the MBOAT7 transcript was significantly downregulated after lipopolysaccharide stimulation (Fig. S1A), an effect that was completely reversed by a TLR4 inhibitor (TAK-242) that we confirmed abrogated TLR4 induced TNF-α (Fig. S1B). Using TLR4 small interfering RNAs (siRNAs), we confirmed the specificity of this effect Fig. S1C). Given the association of rs8736 in *MBOAT7* in MAFLD^11^, we examined if MBOAT7 was expressed and regulated by TLRs in human Kupffer cells as well. Consistently, MBOAT7 expression was comparable to that in MDMs and similarly attenuated upon TLR4 stimulation (Fig. S1D) and also by Western blot (Fig. S1E).

Notably, stimulation of cells with a pro- (IFN-γ and IL-β) or anti-inflammatory cytokine (interleukin-4 [IL-4]) did not modify MBOAT7 expression (Fig. S1F) indicating that the expression of MBOAT7 is specifically altered by TLRs signalling. We next focused on the contributions and mechanisms for MBOAT7-mediated regulation of TLRs-initiated outcomes in primary human cells given the association of MBOAT7 with human disease.

### MDMs from rs8736 T risk carriers demonstrate lower MBOAT7 expression and increased TLRs-induced cytokine secretion

The rs8736 polymorphism (LD to rs641738T: *r*^2^ = 0.98) in *MBOAT7* is associated with MAFLD^11^ and TLRs specifically downregulate MBOAT7 in MDMs. Hence, we questioned if MBOAT7 expression in response to TLRs signalling is regulated in a genotype-dependent manner. To this end, we stimulated MDMs from healthy individuals and genotyped them for rs8736. Persistent inflammation is a major determinant of MAFLD progression^21^. Therefore, to avoid the confounding effects of a chronic inflammatory state and/or medications in patients, we studied the risk allele in healthy individuals. To do this we used an allele-specific assay to analyze expression levels of each allele (C or T) in persons heterozygous for the MAFLD-associated SNP (rs8736) in unstimulated conditions and after TLRs stimulation. By examining mRNA expression from heterozygous subjects we could assess the expression of each allele within an individual donor, thus ensuring identical stimulation conditions. MDMs from rs8736 TT carriers expressed less MBOAT7 mRNA than CC carriers at baseline and upon TLR4 stimulation (Fig. S1G). We considered if the gene TMC4 near the MBOAT7 locus may be accounting for the genotype-dependent effects observed. However, this gene was not detected in MDMs.

**Fig. 1:**
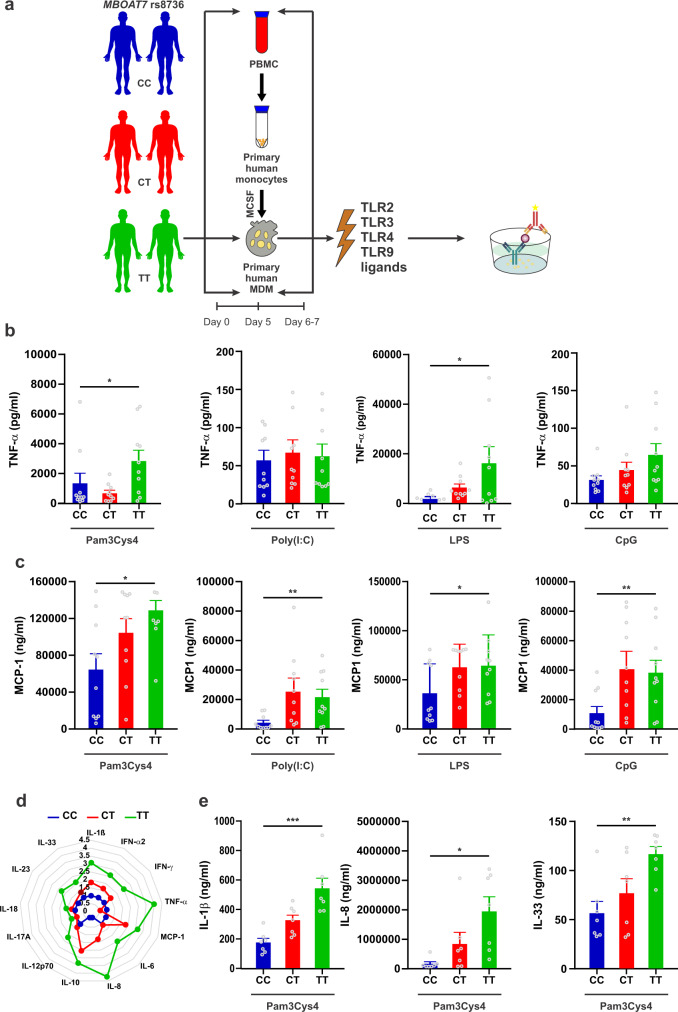
Human macrophages from rs8736 TT carriers demonstrate higher cytokine secretion upon TLR stimulation compared with C allele carriers. **a** Scheme illustrating the experimental design. MDMs (*n* = 10/genotype) were treated for 24 h with the indicated TLRs ligands (Pam3Cys4 [TLR2], Poly(I:C) [TLR3], LPS [TLR4], and CpG [TLR9]). Shown is quantitative ELISA of the conditional media measuring **b** TNF-α (*P* = 0.04, 0.8, 0.02, and 0.9, respectively) and **c** MCP-1 (*P* = 0.02, 0.009, 0.04, and 0.01, respectively). **d** Radar chart representing fold increase of different chemokines and cytokines; values segregated by donor genotype secreted by MDMs (*n* = 7/genotype) were treated for 24 h with Pam3Cys4 [TLR2]. **e** Individual results of IL-1β, IL-8 and IL-33 (*P* = 0.0007, *P* = 0.01, *P* = 0.005, respectively). Data are represented by vertical bars and are mean ± sem; *P < 0.05, ** *P* < 0.01, ****P < 0.001. Statistical differences between groups were assessed by one-way ANOVA; multiple comparisons were corrected by Bonferroni correction. Source data are provided as a Source Data file.

TLRs-initiated outcomes including cytokine secretion are important in MAFLD pathophysiology^1^. We next questioned if TLRs-initiated cytokine secretion from MDMDs is modulated by MBOAT7 genotype (Fig. 1a). Consistent with the lower MBOAT7 expression in MDMs from rs8736 TT compared to CC carriers, MDMs from rs8736 T risk carriers secreted more TNF-α upon TLR4 stimulation than CC carriers (Fig. 1b). Similar upregulation was observed for the proinflammatory cytokine MCP-1 (Fig. 1c). Also, upon stimulation of other TLRs, relative to CC carriers, MDMs from rs8736 T risk carriers secreted more TNF-α and MCP-1 (Fig. 1b, c).

We examined the production of other proinflammatory cytokines to determine whether these also differed by genotype. We quantified 13 human inflammatory cytokines/chemokines, including IL-1β, IFN-α2, IFN-γ, TNF-α, MCP-1, IL-6, IL-8, IL-10, IL-12p70, IL-17A, IL-18, IL-23, and IL-33; as well as producing more TNF-α and MCP-1, MDMs from minor (T) allele homozygotes produced greater IL-1β, IFN-α2, IFN-γ, IL-6, IL-8, IL-12p70, IL-18, IL-23, and IL-33 compared to those from major (C) allele homozygotes upon TLR2 stimulation (Fig. 1d, e and Fig. S1H).

Taken together, MDMs from healthy human subjects harbouring a MAFLD-associated rs8736 (T) risk allele express less MBOAT7 mRNA following activation with a broad range of TLRs. Reduced MBOAT7 expression renders the MDMs hyper-responsive to TLR-induced inflammatory responses, consistent with a role for this SNP in exacerbation of inflammation in MAFLD^11^.

### MBOAT7 expression by the rs8736 variant accounts for MBOAT7-dependent TLR-induced cytokine secretion

We next sought to establish if modulation of MBOAT7 expression accounted for *MBOAT7* genotype-dependent regulation of TLR-induced cytokines. To do this, we reduced the levels of MBOAT7 in MDMs and confirmed mRNA and protein reduction for the knockdown studies (Fig. S2A). Knock down of MBOAT7 in MDMs did not affect cell viability or apoptosis (Fig. S2B), or the expression of other MBOAT family genes (Fig. S2C). The cells remained responsive to alternative stimuli, which excluded cell death as an explanation for the effects observed on inflammation-related gene expression.

**Fig. 2:**
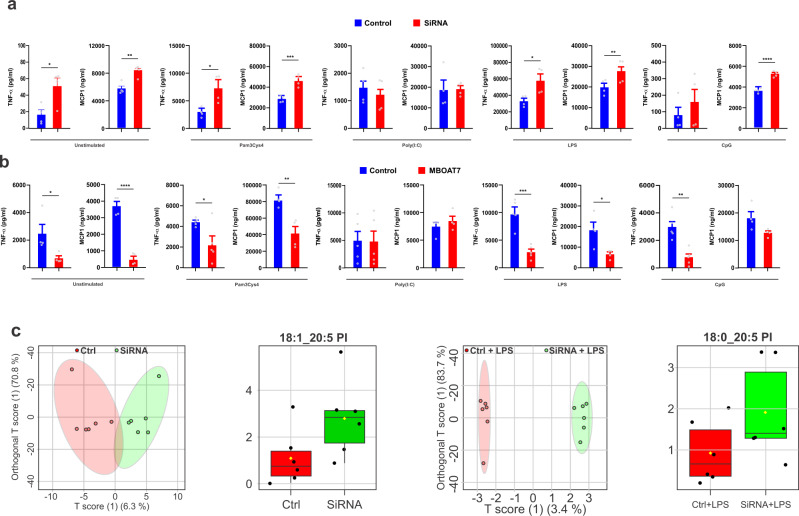
MBOAT7 is required for optimal secretion of cytokines and remodelling of phospholipids in MDMs. Quantitative ELISA of the conditional media measuring TNF-α and MCP-1in Unstimulated MDMs and cells treated with the indicated TLRs ligands (Pam3Cys4 [TLR2], Poly(I:C) [TLR3], LPS [TLR4] and CpG [TLR9]) for 24 h in **a** MDMs (*n* = 4–5) were transfected with MBOAT7 siRNA or scramble as control, **b** MDMs (*n* = 4–6) were transfected with MBOAT7 overexpression plasmid or the empty vector as control. *P*-value <0.02 for all comparisons except for TLR3. **c** LC-MS/MS analysis of phospholipid species in MDMs transfected with MBOAT7 siRNA or scramble at base line and challenged with LPS for 24 h (*n* = 6). Statistical differences between groups were assessed by 2-tailed Student’s t test and the error bars indicate the mean ± sem; *P < 0.05, ** *P* < 0.01, ****P < 0.001. Box plots indicate the first and third quartiles with the median in between, the whiskers extend to the furthest point within 1.5× the inter-quartile range past the corresponding quartile. Source data are provided as a Source Data file.

With reduction of MBOAT7 expression, cells demonstrated higher expression levels of inflammatory cytokines (Fig. S2F) and higher levels of TNF-α and MCP-1 compared with control cells upon stimulation of a broad range of TLRs (TLR2, TLR4, and TLR9), but not TLR3, the only TLR not to use MyD88 for intracellular signalling^22^ (Fig. 2a). The results were confirmed with MBOAT7 knockdown with 2 additional siRNAs (Fig. S2G), as well as in Kupffer cells (Fig. S2H).

We conducted complementary studies wherein MBOAT7 expression was increased in MDMs (Fig. S2D); this did not impact the expression of other MBOAT family genes (Fig. S2E) but consistently led to lower levels of cytokine mRNA expression (Fig. S2I) and TNF-α and MCP-1 secretion at baseline and upon TLRs stimulation when compared with control cells (Fig. 2b).

We tested whether MBOAT7 similarly regulated the TLR response in macrophages cultured with GM-CSF instead of M-CSF. In GM-CSF-cultured macrophages, MBOAT7 knockdown increased TLR4-induced inflammatory cytokine expression (Fig. S2J), similar to M-CSF-cultured macrophages. Therefore, MBOAT7 is required for optimal responses upon stimulation of a broad range of TLRs.

### The rs8736 variant alters MBOAT7 expression through miRNA-dependent mechanisms

**Fig. 3:**
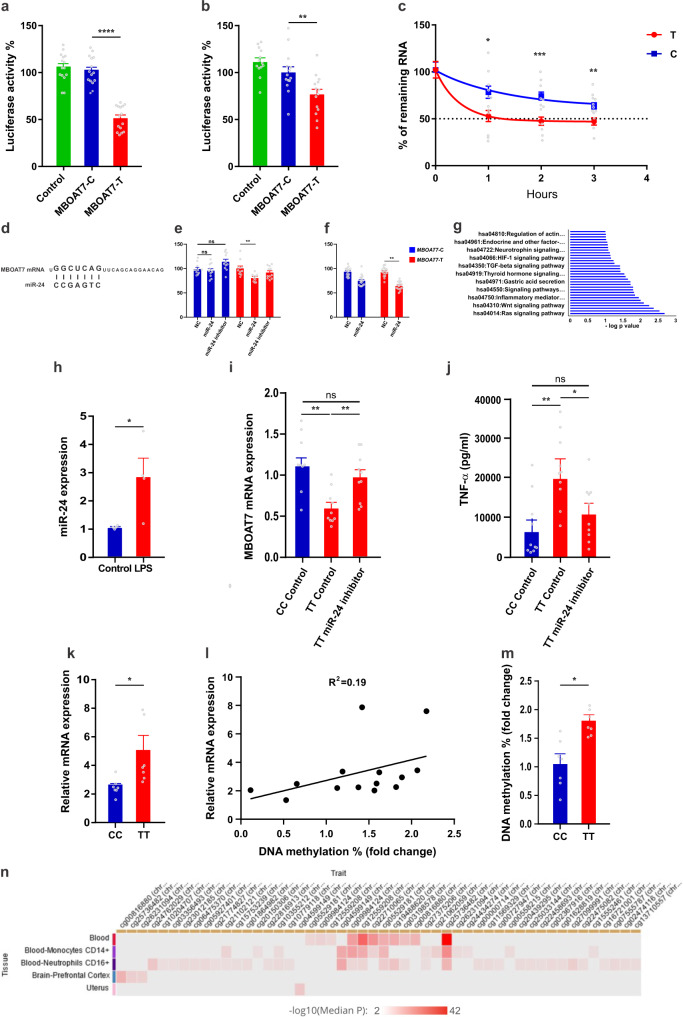
The rs8736 variant alters MBOAT7 expression through miRNA-dependent mechanisms. Luciferase activity in **a** Huh-7 cells and **b** HEK293 cells transfected with full-length 3′UTRs of MBOAT7 containing either the C or the T allele (*n* = 15 and 13 includes 3 biological replicates and 4–5 technical replicates, respectively). **c** Huh-7 cells transfected with the full-length 3′UTRs of MBOAT7 containing either the C or the T allele were treated with actinomycin D and analyzed for stability of MBOAT7 mRNA (*n* = 6 includes three biological replicates and 2 technical replicates). **d** Diagram represents the miRNA-24 binding site in the 3′ UTR of MBOAT7. Luciferase activity in **e** Huh-7 and **f** HEK293 cells double transfected with full-length 3′UTRs of MBOAT7 containing either the C or the T allele and either (miRNA-24 mimic, inhibitor or control) (*n* = 11 and 16 includes 3 biological replicates and 3–5 technical replicates, respectively). **g** Pathway analysis of the common predicted targets of miRNA-24 using KEGG pathway mapping. **h** miRNA-24 relative mRNA expression assessed by q-PCR and normalized to GAPDH in MDMs isolated from subjects heterozygous for rs8736 and challenged with LPS for 24 h (*n* = 9). MDMs from subjects harbouring the CC or the TT genotype for rs8736 and transfected with either miRNA-24 inhibitor or control (*n* = 10/group) **i** MBOAT7 relative mRNA expression assessed by q-PCR and **j** ELISA of the medium measuring TNF-α. **k** Transcripts with 3′-UTRs of different lengths were identified by real-time qPCR in MDMs from subjects harbouring rs8736 CC and TT genotype (*n* = 7/group) and the distal transcript-to-total transcript ratios are shown. **l** The correlation of MBOAT7 distal sites in MDMs to the blood DNA methylation. **m** Increased blood MBOAT7 DNA methylation in subjects harbouring rs8736 TT compared to those with CC genotype (*n* = 7). **n** Heatmap of mQTL for the rs8736 as a variant in different tissues. *P*-value <0.04 for all comparisons. Statistical differences between groups were assessed by two-tailed Student’s t test or one-way ANOVA; multiple comparisons were corrected by Bonferroni correction and the error bars indicate the mean ± sem; *P < 0.05, ** *P* < 0.01, ****P < 0.001. Source data are provided as a Source Data file.

As MBOAT7 modulation accounted for the rs8736 genotype-dependent effects on TLRs-induced cytokines, we sought to understand the underlying mechanisms. We generated full-length 3′UTRs of MBOAT7 containing either the C or the T allele and cloned them into the pmirGLO vector, then transfected human Huh-7 cells with the constructs and measured luciferase activity. The MBOAT7-T 3′UTR conferred significantly lower luciferase activity compared to the MBOAT7-C 3′UTR (Fig. 3a); we confirmed this finding in the human HEK293 cell line (Fig. 3b).

We next assessed the effect of rs8736 on mRNA stability. Analysis of the luciferase–encoding mRNA that remained in Huh-7 cells after treatment with actinomycin D revealed that mRNA bearing the MBOAT7-T 3′UTR decayed significantly faster than that bearing the MBOAT7-C 3′UTR (Fig. 3c). These data imply that the rs8736 allele affects the stability of the mRNA transcript.

Gene expression can be dramatically modulated by miRNA binding and there is a putative miRNA-24 binding site in the 3′ UTR of MBOAT7 (Fig. 3d). When the MBOAT7 3′ UTR construct was expressed in Huh-7 cells along with miRNA-24, luciferase expression from the MBOAT7 3′UTR was reduced (Fig. 3e) and similarly in the HEK293 cell line (Fig. 3f), whereas with the miRNA-24 hairpin inhibitor, luciferase expression was increased (Fig. 3e). Pathway analysis of the common predicted targets of miRNA-24 using KEGG pathway mapping showed that miRNA-24 targets mapped to different pathways including inflammatory ones (Fig. 3g). In addition, LPS upregulated miRNA-24 expression in MDMs (Fig. 3h).

To establish the role of miRNA-24 in the differential regulation of MBOAT7 rs8736 expression, we conducted complementary studies using an miRNA-24 hairpin inhibitor that increased MBOAT7 expression levels in rs8736 TT carrier MDMs to the levels observed in CC carriers (Fig. 3i); this resulted in similar levels of cytokine secretion compared with CC carrier MDMs (Fig. 3j). Therefore, modulation in MBOAT7 expression levels associated with the rs8736 variant accounts for the regulation of the MBOAT7-dependent outcomes observed.

Since the pathological role of the MBOAT7 variant relied on miRNA-mediated silencing leading, we uncovered the mechanisms of this silencing. We hypothesized that alternative polyadenylation mediated different 3′UTR lengths in a rs8736 genotype-dependent manner and altered the response of MBOAT7 mRNA to microRNAs. To this end, we analyzed the ratio of distal/total isoforms in the TT compared to the CC genotype and found it be significantly increased in subjects harbouring the former (Fig. 3k).

Recent findings suggest that DNA methylation regulates alternative polyadenylation.^23^. Therefore, we examined the correlation between the distal isoform of MBOAT7 and the level of DNA methylation in MBOAT7. Our data showed that both are positively correlated (Fig. 3l). Finally, we questioned if the rs8736 T risk variant demonstrates higher DNA methylation than the rs8736 C variant and found this to be the case (Fig. 3m). Similarly, using QTLbase annotation, the SNP variant is also mQTL in blood and monocytes (Fig. 3n).

Taken together, our results demonstrate that rs8736 can alter usage of the distal poly(A) site via DNA methylation and thereby regulates miRNA-mediated silencing. This plays a decisive role in mRNA stability and dictates the final expression of MBOAT7.

### Depletion of MBOAT7 in macrophages results in remodelling of phospholipids

We considered how MBOAT7 might be modulating inflammatory cytokines production. MBOAT7 is known to catalyze the selective esterification of arachidonyl-CoA to LPI lipids in neutrophils^14^. Hence, we examined the impact of MBOAT7 on phospholipids in MDMs. MBOAT7 knockdown led to profound remodelling of PI species at baseline and upon TLR4 stimulation with accumulation of 20:5-containing PI species (Fig. 2c) levels in the MDMs. Silencing of MBOAT7 had no effect on other lipid species such as cholesterol (Fig. S3A) or triglycerides (Fig. S3B).

MBOAT7 through its role in arachidonate recycling regulates free arachidonic acid-derived immunomodulatory lipid mediators^14^. Eicosanoids are formed when phospholipase A2 (PLA2) action liberates arachidonic acid from the sn-2 position of membrane phospholipids. Free arachidonic acid is then converted into potent bioactive mediators by the action of various cyclooxygenases (COX) and lipoxygenases (LOX) (Fig. S3C). Therefore, we hypothesized that MBOAT7 knockdown might expand the pool of arachidonic acid-derived pro-inflammatory eicosanoids. Consistently, we observed a decrease in arachidonic acid in MBOAT7 knockdown cells compared to controls at baseline, though this difference was less clear upon stimulation with TLR4 (Fig. S3D). In addition, in the cyclooxygenases (COX) pathway, PGE2 and in the 5-LOX pathway, 12-HETE were elevated (Fig. S3E). The pro-resolving n-3 fatty acids eicosapentaenoic acid (EPA) and docosahexaenoic acid (DHA) were down regulated in MBOAT7 knockdown cells compared to control (Fig. S3F).

We measured if MBOAT7 inhibition impacts the activity of this pathway. MBOAT7 inhibition led to increased LOX activity at baseline and on stimulation with TLR4 (Fig. S3G), and COX activity upon stimulation with TLR4 (Fig. S3H), while it has no effect on PLA2 activity (Fig. S3I).

We investigated if MBOAT7 deficiency-induced eicosanoids production is responsible for the elevated cytokine production. To this end, we tested the impact of pharmacological inhibitors of key enzymes in the eicosanoids production pathway, namely phospholipase A2 (PLA2) (AACOCF3)^24^, 12/15-LOX (PD146176), 5-LOX (L655)^25^ and the pan-COX inhibitor indomethacin^26^. Inhibition of key enzymes in the eicosanoids production pathway did not have a profound impact on cytokine secretion (Fig. S3J). Collectively, these data support a rate-limiting role for MBOAT7 in arachidonic acid-derived lipid mediator production in MDMs. While eicosanoids are partially involved in the pro-inflammatory effect of MBOAT7 depletion, other mechanisms likely mediate this effect.

### MBOAT7 deficiency induces ER stress

**Fig. 4:**
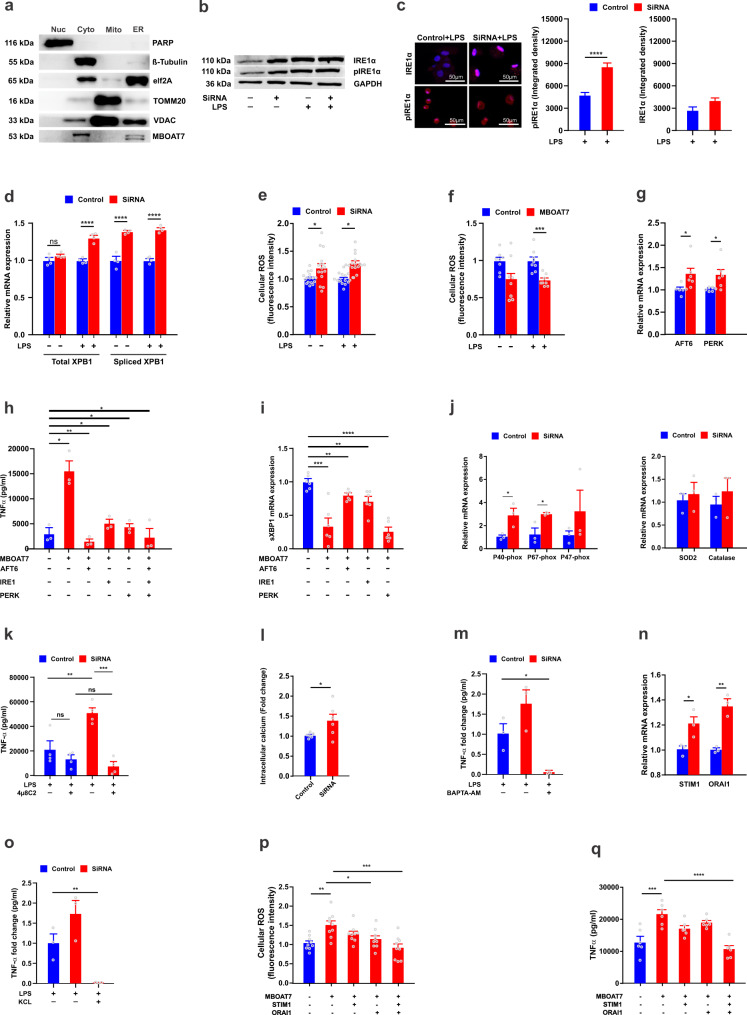
MBOAT7 deficiency induces ER stress. **a** Western blot analysis of MBOAT7 expression in subcellular fractions of THP-1 cells. Phosphorylation of ER stress marker, IRE1A, analyzed by **b** immunoblot and **c** immunofluorescence in MDMs transfected with MBOAT7 siRNA or control with and without LPS treatment (*n* = 3). **d** Induction of Xbp-1 splicing analyzed by PCR and normalized to GAPDH in MDMs transfected with MBOAT7 siRNA or control with and without LPS treatment (*n* = 4). Total cellular ROS production with and without LPS stimulation in **e** knockdown and **f** overexpression MBOAT7 in MDMs (*n* = 16 and 8 includes 3 and 2 biological replicates from independent individuals and 4–5 technical replicates, respectively). **g** Real-time PCR for the relative mRNA expression of UPR branches in MDMs transfected with MBOAT7 siRNA or scramble as control. Effect of inhibition of UPR branches on **h** TNF-α secretion assessed by ELISA and **i** sXBP1 mRNA expression. **j** Real-time PCR of NADPH complex and antioxidant enzymes in MDMs transfected with MBOAT7 siRNA or scramble (*n* = 3). **k** Effect of 4µ8C (IREA1 inhibitor) on TNF-α secretion assessed by ELISA in MDMs knocked-down for MBOAT7 and challenged with LPS (*n* = 4). **l** Intracelluar Ca2^+^ levels in in MDMs transfected with MBOAT7 siRNA or scramble after stimulation with LPS. **m** LPS-induced TNF-α secretion from MBOAT7 deficient cells was suppressed by the intracellular Ca2^+^-chelating agent BAPTA-AM and **o** high extracellular K^+^ (*n* = 3). **n** Expression levels of TRPC1, Orail1 and STIM1 in control and MBOAT7 depleted cells (*n* = 3). MDMs were transfected with scramble, MBOAT7 siRNA, STIM1siRNA, Orail1 siRNA, or a combination of the three siRNAs for 48 h and treated with LPS for 4 h. Shown is **p** total cellular ROS (*n* = 9 includes three biological replicates from independent individuals and three technical replicates) and **q** TNF-α secretion in MDMs. (*n* = 6 includes three biological replicates from independent individuals and two technical replicates in **g**–**i**, **l**, and **q**). *P*-value <0.04 for all comparisons. Statistical differences between groups were assessed by two-tailed Student’s t test and the error bars indicate the mean ± sem; *P < 0.05, ** *P* < 0.01, ****P < 0.001. Source data are provided as a Source Data file.

To further define the mechanisms for MBOAT7 contributions to TLRs-induced signalling, the intracellular localization of MBOAT7 was evaluated in the human macrophage PMA-differentiated THP-1 cell line. As previously reported in hepatocytes^11^, MBOAT7 co-localized with purified endoplasmic reticulum (ER) membranes, but not with mitochondria (Fig. 4a). Considering the regulatory effect of membrane remodelling and ER stress on macrophage activation^27^, we speculated that MBOAT7 might modulate ER-dependent macrophage activation providing an alternative mechanism to explain the enhanced inflammatory activation associated with MBOAT7 deficiency.

We first established that in human MDMs, TLR4 activated the ER stress pathway. We demonstrated that transcripts of IRE1α and spliced XBP1, occurring downstream of the IRE1α pathway, and ERdj4, and BiP, additional ER-stress induced transcripts^28^ were induced after TLR4 treatment (Fig. S4A). This was confirmed by western blot, while LPS treatment activated IRE1α (Fig. S4B).

We next assessed if MBOAT7 regulates TLR-induced ER stress. As expected, upon TLR stimulation of MDMs, MBOAT7 knockdown resulted in increased ER stress markers mRNA expression (Fig. S4C). This was confirmed for IREA1α by Western blot (Fig. 4b) and by immunofluorescence (Fig. 4c). MBOAT7 depletion also increased spliced XBP1 (Fig. 4d) and total cellular ROS production using the fluorescent probe 2′7′-dichlorofluorescin diacetate (DCF-DA) (Fig. 4e). In complementary studies, increased MBOAT7 expression in MDMs decreased ER stress markers (Fig. S4D) and ROS production (Fig. 4f).

We then sought to address mechanisms through which MBOAT7-dependent ER stress contributes to TLRs-induced outcomes. To this end, we knocked down each ER stress branch and confirmed that knockdown of each resulted in a selective decrease of the targeted transcript while cell survival remained intact. We observed cross-talk between the three UPR branches as the other two branches were upregulated in response to loss of one branch (Fig. S4E). MBOAT7 knockdown led to the induction of all UPR branches (Fig. 4g) and consistent with the early time at which ER stress is observed, all three branches of ER stress were required for TLR4-induced cytokine production in MBOAT7 depleted cells (Fig. 4h) and the expression of spliced XBP1 (Fig. 4I); combined blockade led to an even greater reduction.

Pro-inflammatory conditions may themselves be activating all three branches of ER stress and contribute to XBP1 activation. We treated MDM with TNF-α and found that it activated UPR branches and induced ER-stress response transcripts (Fig. S4F). Taken together, each of the ER-stress branches is required for TLR4-induced signalling and cytokine production in MBOAT7 depleted cells. The accompanied induction of cytokines might contribute to the activation of each of the ER-stress branches.

To further define mechanisms for the increase in ROS production with MBOAT7 depletion, we examined members of the NADPH complex required for cellular ROS and found induction of p40phox, p47phox, and p67phox mRNA with MBOAT7 knockdown (Fig. 4j). In contrast, the expression of the antioxidant enzymes catalase and SOD2 was not affected by MBOAT7 deficiency (Fig. 4j). This observation indicates that the increase in ROS levels is due to an increase in ROS production rather than to decreased degradation.

To address the role of the ER stress pathway downstream of MBOAT7, we pretreated MBOAT7 knockdown and control cells with 4µ8C (an IREA1 inhibitor)^29^ before challenging with TLR4. Inhibition of ER stress led to a drastic reduction of inflammatory cytokines secretion (Fig. 4k) and TLR4-induced transcript upregulation (Fig. S4G), with minimal impact on control cells.

Recently, it has become apparent that a feed-forward vicious cycle and self-amplifying loop between ROS and calcium is an essential mediator that links ER stress to inflammation and cytokine production^30^. Thus, to further delineate in-depth the linking mechanisms between MBOAT7 function and changes in ROS and cytokine production, we proceeded to investigate if MBOAT7 depletion alters intracellular levels of Ca2^+^. We verified that upon MBOAT7 silencing, cells display an increase in intracellular Ca2^+^ concentration assessed using a Ca2^+^-sensitive dye, Fluo-8 (Fig. 4l). To investigate the possible role of intracellular Ca2^+^ in the induction of cytokines generation in MBOAT7 depleted cells, we used the Ca2^+^ chelator 1,2-bis(o-aminophenoxy)ethane-N,N,N′,N′-tetraacetic acid (BAPTA) and found that this severely compromised cytokines generation (Fig. 4m). To further define the mechanisms for the increase in intracellular Ca2^+^, we reasoned that this would be due to activating Ca2^+^ channels. Therefore, we measured the expression levels of Orail1 and STIM1 in control and MBOAT7 depleted cells. Silencing of MBOAT7 significantly increased their expression level (Fig. 4n). K^+^ efflux can regulate Ca2^+^ flux by acting as a counter ion at the plasma membrane for Ca2^+^ influx, and they are coordinated^31^. Thus, we examined the impact of K^+^ efflux and found that cytokines generation in MBOAT7 depleted cells is strongly reduced in the presence of a high extracellular K^+^ concentration (Fig. 4o). For further confirmation, we knocked down Ca2^+^ channels (Orail1 and STIM1). This attenuated MBOAT7 depletion-induced induction of ROS (Fig. 4p) and inflammatory cytokines secretion (Fig. 4q); combined blockade led to an even greater effect.

Collectively, these findings indicate that increased ROS production and calcium levels represents an important mechanism underlying the augmentation in pro-inflammatory activation of MBOAT7 knockdown macrophages.

### The MBOAT7 rs8736 risk variant shows increased TLRs-induced ER stress and downstream outcomes

Having identified a role for MBOAT7 deficiency in increasing TLR-induced ER stress in human MDMs, we questioned whether the MBOAT7 rs8736 variant regulates these effects. Compared to MDMs from subjects carrying the C allele, MDMs from those harbouring the TT genotype showed a significant increase in ER stress-related transcripts (Fig. S4I). For confirmation, we transfected plasmids expressing the C and T allele into HeLa cells and stimulated cells with TLR4 for 4 h. Consistently, the T allele showed an increase in each of the TLR4-induced ER stress-related transcripts (Fig. S4J), ROS (Fig. S4K), inflammatory marker expression (Fig. S4L) and TNF-α secretion (Fig. S4M). Taken together, the MBOAT7 inflammation-risk variant demonstrates increased TLRs-induced ER stress and ER-stress-associated outcomes.

### MBOAT7 deficiency alters mitochondrial function

**Fig. 5:**
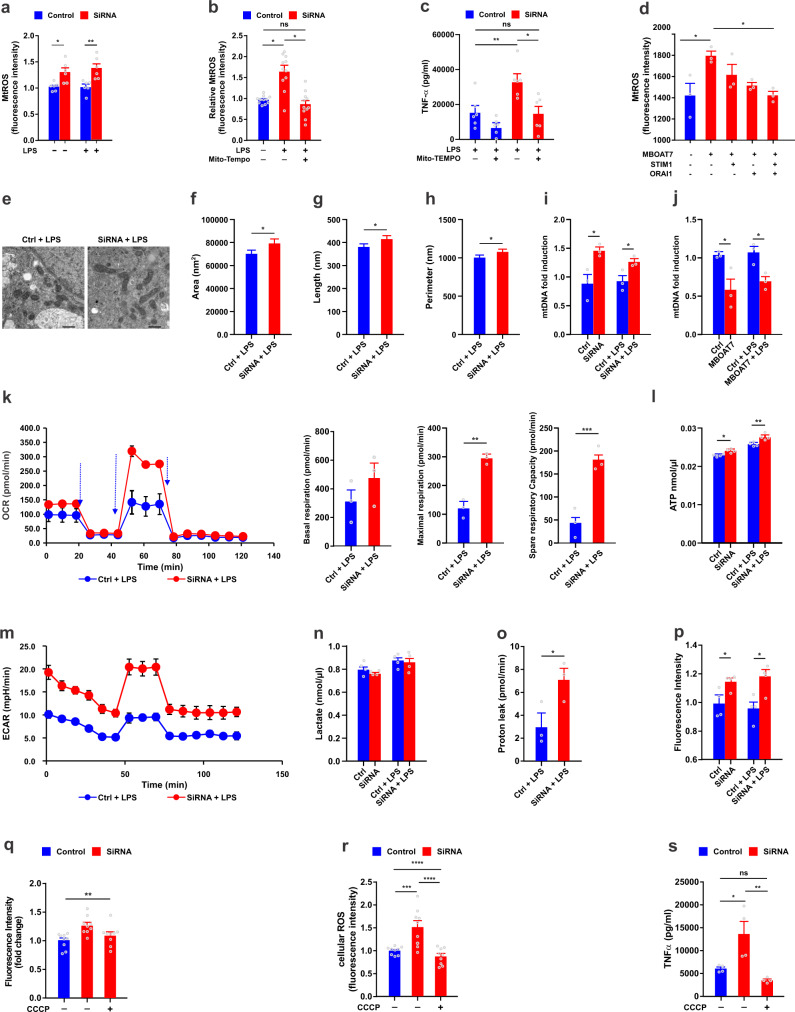
MBOAT7 deficiency alters mitochondrial function. MDMs transfected with MBOAT7 siRNA or scramble as control and treated with LPS for 24 h. **a** Mitochondrial ROS (mROS) measured by the red fluorescent dye MitoSox in MDM transfected with MBOAT7 siRNA or control under basal conditions and after LPS stimulation (*n* = 6). Impact of Mito-TEMPO on **b** mROS (*n* = 11 includes three biological replicates from independent individuals and 3–4 technical replicates) and **c** TNF-α secretion in MDMs (*n* = 6). **d** MDMs were transfected with non-coding scramble, MBOAT7 siRNA, STIM1siRNA, Orail1 siRNA, or a combination of the three siRNAs for 48 h and treated with LPS [TLR4] for 4 h (*n* = 3); mROS is shown. **e** Representative TEM imaging (*n* = 20 images in total) of mitochondria in Scale bar, 500 nm. Over 100 mitochondria in each group were quantified for **f** area, **g** length, and **h** perimeter. Relative induction of mitochondrial DNA (mtDNA) was measured by qRT-PCR in MDMs at baseline and when challenged with LPS for 24 h after transfection with **i** MBOAT7 siRNA or scramble as control and **j** MBOAT7 overexpression plasmid or the empty vector as control (*n* = 3). In MDMs knocked down for MBOAT7 unstimulated or stimulated with LPS (*n* = 3–4); **k** Extracellular flux analyzer of oxygen consumption rate (OCR) obtained over time (min), **l** mitochondrial ATP levels, **m** extracellular acidification rate (ECAR) obtained over time (min), **n** lactate levels and **o** quantification of proton leak, **p** mitochondrial membrane potential quantified by fluorescence intensity. Short treatment of MDMs with the depolarizing agent carbonyl cyanide m-chlorophenyl hydrazone (CCCP) for 1 h and shown is the effect of CCCP in MDMs knocked-down for MBOAT7 and challenged with LPS on **q** Mitochondrial membrane potential quantified by fluorescence intensity, **r** total cellular ROS, and **s** TNF-α secretion assessed by ELISA (*n* = 4). (*n* = 9 includes three biological replicates from independent individuals and three technical replicates in **q** and **r**). *P*-value <0.04 for all comparisons. Statistical differences between groups were assessed by two-tailed Student’s t test and the error bars indicate the mean ± sem; *P < 0.05, ** *P* < 0.01, ****P < 0.001. Source data are provided as a Source Data file.

ER and mitochondria are interdependent organelles and ER stress induces mitochondrial damage^32^. Mitochondria are a principal source of cellular ROS but are also increasingly recognized as central hubs for innate immune signalling^33–35^. Thus, we explored if MBOAT7 might modulate mitochondria dependent macrophage activation. In addition to total cellular ROS, in the absence of MBOAT7, the production of mitochondrial ROS (mROS) in MDMs was increased under basal conditions and after TLR4 stimulation (Fig. 5a). Mito-TEMPO, a specific scavenger of mitochondrial ROS^36^ attenuated MBOAT7 depletion induced induction of mROS (Fig. 5b), inflammatory cytokines secretion (Fig. 5C), and TLR4-induced transcript upregulation (Fig. S4H). In addition, knock down of Ca2+ channels (Orail1 and STIM1) attenuated MBOAT7 depletion induced induction of mROS, while combined blockade had an even greater effect (Fig. 5d).

To assess the impact of MBOAT7 deficiency on mitochondrial morphology, we applied transmission electron microscopy (TEM) which show that MBOAT7 knockdown macrophages stimulated with LPS have increased mitochondrial length and perimeter (Fig. 5e–h). Moreover, the higher mtDNA/nDNA ratio confirmed that MBOAT7 knockdown increases mtDNA copy number compared to control cells (Fig. 5i). Increasing MBOAT7 expression leads to the reverse effect (Fig. 5j).

Mitochondrial shape and bioenergetics are intimately linked^37^. Thus, we used an extracellular flux assay, which allows for direct evaluation of cellular bioenergetic profiles by measuring the oxygen consumption rate (OCR, measure of oxidative phosphorylation) and extracellular acidification rate (ECAR, measure of aerobic glycolysis). Unexpectedly, both the basal and maximal respiratory capacity (OCR) (Fig. 5k) in MBOAT7 deficient cells generated more mitochondrial ATP (Fig. 5l) and aerobic glycolysis (ECAR) (Fig. 5m) was increased in knockdown cells compared to controls, but with no significant effect on lactate levels (Fig. 5n). Consistently, MBOAT7 knockdown led to increased proton leak (Fig. 5o), which likely represents a compensatory mechanism to restore adaptation and decrease ROS, as previously reported^38^. In addition, MBOAT7 deficient cells displayed a higher mitochondrial membrane potential (Fig. 5p). Interfering with MMP via short-term treatment of MDMs with the depolarizing agent carbonyl cyanide m-chlorophenyl hydrazone (CCCP) had no impact on cell viability, and restored the mitochondrial membrane potential to the control level (Fig. 5q). This significantly reduced the MBOAT7 depletion-induced induction of ROS (Fig. 5r) and inflammatory cytokines secretion (Fig. 5s). This data indicates that the increased pro-inflammatory effects of MBOAT7 knockdown are dependent on an increased MMP.

Next, we explored the molecular mechanisms of MBOAT7 depletion in increasing OXPHOS during inflammation. To do this, we measured mRNA levels of the biogenesis-related factors SIRT1, NRF1, and TFAM in the macrophages; they were not changed (Fig. S4N). ATP production in mitochondria relies on the electron transport chain and oxidative phosphorylation^39^. This process involves five multi-subunit respiratory complexes (I–V). We analyzed the transcription of specific components of these complexes by qPCR. The mRNA levels of UQCRC2 (complex III), MT-CO1 (complex IV), and ATP5A1 (complex V), but not NDUFB8 (complex I) and SDHB (complex II) were significantly increased in response to MBOAT7 knockdown (Fig. S4N).

We also considered the possibility that MBOAT7 regulates OXPHOS during inflammation by altering mitochondrial dynamics. Thus, we examined the expression levels of several critical regulators^40,41^. We found higher expression levels of the mitochondrial fusion regulators MFN1/MFN2 and FAM73b in MBOAT7-depleted cells compared to controls (Fig. S4N). We did not observe any difference in the expression of the positive regulators of mitochondrial fission including dynamin-related protein 1 (DRP1), STAT2, and mitochondrial elongation factor 1 (MIEF1) (Fig. S4N).

Collectively, these data indicate that MBOAT7 functions as a critical regulator of mitochondrial dynamics during TLR stimulation. MBOAT7 deficiency in MDMs reconfigures energy metabolic pathways and results in an energy-consuming phenotype (high respiratory capacity, high glycolysis). This supports a hyper-inflammatory status in macrophages and provides the required energy for cytokine production.

### MBOAT7 Limits NLRP3 inflammasome activation

Activation of the Nod-like receptors 3 (NLRP3) inflammasome requires two signals. The first signal can be elicited by TLR ligands or TNF-α whereas numerous molecules can provide signal 2 such as ATP^42^. A well-characterized outcome of TLR and inflammasome cooperation is IL-1β secretion. IL-1β is translated as pro-IL-1β that stays in an inactivated form until a second signal activates inflammasomes leading to pro-IL-1β cleavage by caspase-1 to a mature form that is secreted^42^. mtROS production and calcium flux act as a central trigger that activates the NLRP3 inflammasome. Thus, we investigated whether MBOAT7 controls NLRP3 inflammasome activation. Secreted IL-1β levels were significantly increased on silencing MBOAT7 (Fig. S5A). In contrast, MBOAT7 deficiency had no effect on the secretion of IL-1β in response to the NLRP1 inflammasome activator muramyl dipeptide (MDP), the NLRC4 inflammasome activator flagellin, or the AIM2 inflammasome activator poly(dA:dT) (Fig. S5B) indicating the specific effects of MBOAT7 on the NLRP3 inflammasome. Furthermore, MBOAT7 silencing led to higher levels of active caspase-1 (Fig. S5C) and protein levels of pro- IL-1β and NLRP3 (Fig. S5D) in LPS-primed MDMs in response to ATP. Consistently, MBOAT7 activation attenuated secreted IL-1β levels (Fig. S5E).

Next, we investigated whether the MBOAT7 rs8736 variant regulates these effects. Compared to MDMs from subjects carrying the C allele, MDMs from those harbouring the TT genotype produced greater secreted IL-1β levels (Fig. S5F). Similarly, the T allele in HeLa cells showed an increase in IL-1β secretion compared to C allele cells (Fig. S5G) though both comparisons did not reach a statistical significance, but had significantly higher IL-B mRNA expression (Fig. S5H). Taken together, MBOAT7 selectively limits NLRP3 inflammasome activation.

### MBOAT7 inhibition promotes remodelling of the accessible inflammatory-related chromatin landscape

**Fig. 6:**
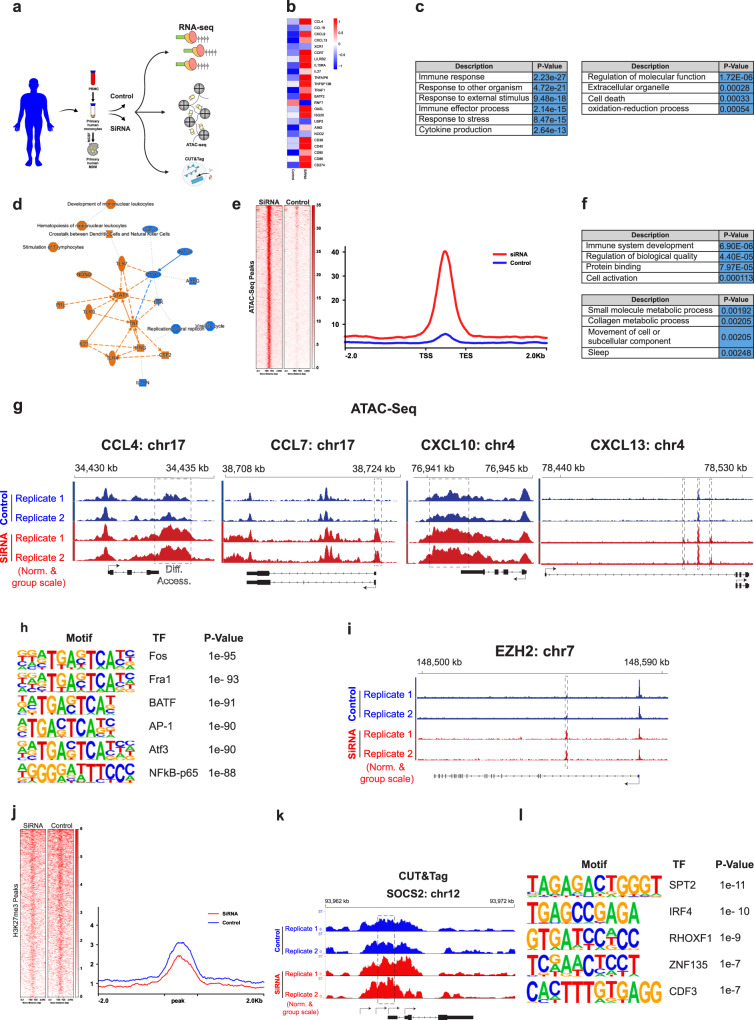
MBOAT7 remodels the inflammation related chromatin landscape and the suppressive histone landscape. **a** Scheme illustrating the experimental design. **b** Heatmap showing relative expression of the subset of inflammatory and immune response genes. **c** Gene ontology categories enriched in the subset of MBOAT7 depletion-induced repressed and induced genes. **d** Ingenuity pathways analysis (IPA, Ingenuity systems). **e** Heatmap of ATAC-seq (±2 kb from peak centre) in MDM transfected with MBOAT7 siRNA or control. **f** Gene ontology categories enriched in the subset of MBOAT7 depletion-induced differential peaks in the ATAC-seq. **g** Browser tracks showing ATAC-seq data at indicated loci. Peaks are highlighted by black boxes. **h** Transcription factor (TF) motifs enriched in chromatin regions showing increased accessibility on MBOAT7 depletion. **i** Browser tracks showing ATAC-seq data at EZH2 locus. **j** Heatmap of H3K27me3 Cut&tag (±2 kb from peak centre) in MDM transfected with MBOAT7 siRNA or control. **k** Browser tracks showing H3K27me3 at SOCS2. **l** TF binding motifs enriched at sites of MBOAT7 depletion-induced differential peaks enrichment in H3K27me3.

Induction of cytokine gene expression downstream of canonical TLR signalling is constrained by a chromatin barrier whose remodelling is required for the expression of many TLR-inducible genes^43,44^. Growing evidence suggests that oxidative stress can induce chromatin remodelling that mediates changes in gene transcription^45^. Thus, to investigate the molecular mechanisms of MBOAT7 in regulating the TLR-induced inflammatory response, we explored the possibility that depletion of MBOAT7 overcomes this chromatin barrier and induces chromatin remodelling that can prime the chromatin for transcriptional activation changes upon TLR stimulation. To do this we have undertaken integrated analysis by RNA-seq (RNA-sequencing), ATAC-seq (Assay for Transposase-Accessible Chromatin using sequencing) and CUT&Tag-seq (cleavage under targets and tagmentation sequencing) in MDMs (Fig. 6a).

RNA-Seq showed upregulation in the expression of TNF-α responsive genes associated with inflammation (e.g., CCL4, CXCL9, TRAF1, CD274, and CCR7), surface markers (e.g., CD38, CD80, and CD86), and the immune response (e.g., OASL, ISG20, AIM2, and NOD2) with MBOAT7 knockdown (Fig. 6b). Analysis based on GO biological function determined that the MBOAT7 depletion-modified genes were mainly enriched for immune responses and cytokine production along with metabolic related signalling in the induced and repressed gene sets, respectively (Fig. 6c). To explore gene networks regulated by MBOAT7 depletion, we performed network analyses derived from the differentially expressed gene list using ingenuity pathway analysis (IPA). This identified enriched networks of genes related to immune and inflammatory responses such as TNF-α, and TLRs (Fig. 6d).

We examined the impact of MBOAT7 depletion on genome-wide chromatin accessibility. Notably, transposase-based sequencing (ATAC-seq) revealed chromatin remodelling upon MBOAT7 knockdown. MBOAT7 depletion increased chromatin accessibility and resulted in increases at more than 567 peaks and decreases in 267 peaks (Fig. 6e). GO analysis of annotated genes associated with the open chromatin regions identified active biological processes including the immune response. In contrast, metabolic-related signalling was noticed with closed chromatin regions (Fig. 6f). In particular, chromatin accessibility at inflammatory cytokines (e.g., CCL4, CXCL3, CXCL10, and CCR7) was increased on MBOAT7 depletion (Fig. 6g), consistent with the RNA-seq data. Motif analyses of MBOAT7 knockdown-induced ATAC-seq peaks identified a set of the AP-1 transcription factor members including Fos, Fra1, BTAF, AP-1, and ATF3 as the top-ranking motifs together with NF-κB (Fig. 6h). This suggests a direct role for MBOAT7 in counteracting the effects of inflammatory cytokines.

Interestingly, MBOAT7 deficiency led to an increase in chromatin accessibility of EZH2 (Fig. 6i) and Suz12, members of the polycomb repressive complex-2 (PRC2) that play crucial roles in trimethylated histone H3 lysine 27 (H3K27me3) mediated repression of genes^46^. EZH2 and H3K27me3 are widely expressed in immune cells and have been reported to facilitate macrophage activation and pro-inflammatory responses via inhibition of suppressors of cytokine signalling^47^. Thus, to better understand how MBOAT7 impacts macrophage inflammatory responses, a genome-wide mapping of the histone methylation landscape was generated using H3K27me3 (Fig. 6j). Depletion of MBOAT7 significantly altered the repressive landscape of a subset of genes. Intriguingly, H3K27me3 was increased for anti-inflammatory genes such as SOCS2 (Fig. 6k) and SOCS3 in MBOAT7-depleted cells. Consistently, motif analyses of MBOAT7 knockdown induced H3K27me3 peaks identified IRF4 as the top-ranking motif together with SPT2 (Fig. 6l). IRF4 is a negative regulator of TLRs and regulates the production of proinflammatory cytokines by macrophages in response to LPS and signals via complexes containing BATF/AP-1 motifs^48,49^.

Collectively, these findings support a model in which MBOAT7 deficiency controls macrophage activation in disease through selective chromatin accessibility by stabilizing “hyper-open” inflammatory regions, epigenetic silencing of select anti-inflammatory pathways, and modulation of transcriptional networks mediated by the combined action of NF-κB, IRF4, and AP-1.

### MDMs from rs8736 T risk carrier patients with MAFLD demonstrate lower MBOAT7 expression and increased TLRs-induced secretion of cytokines

To address whether our findings reflect inflammatory gene regulation in vivo, we generated MDMs from patients with MAFLD and genotyped them for rs8736; their characteristics are depicted in Table S1. MDMs from rs8736 T risk carriers had lower MBOAT7 expression (Fig. S6A) and secreted more TNF-α and MCP-1 (Fig. S6B) upon TLR4 stimulation compared to CC carriers. To confirm the role of miRNA-24 in the rs8736-driven differential regulation of MBOAT7 expression, we used an miRNA-24 hairpin inhibitor that increased MBOAT7 expression levels in rs8736 TT risk–carrier MDMs to the levels observed in CC carrier MDMs (Fig. S6C); this resulted in similar levels of cytokine secretion compared with CC carrier MDMs (Fig. S6D).

### MBOAT7 is downregulated in patients with metabolic steatohepatitis and correlates with inflammation

To relate our findings back to tissues outcomes, we examined MBOAT7 liver expression in control subjects (*n* = 18) and patients with metabolic steatohepatitis (*n* = 38). MBOAT7 mRNA levels were considerably decreased in the livers of patients with steatohepatitis compared to controls (*p* < 0.01) (Fig. S6E). Next, ArrayExpress public repository data (E-MEXP-3291) was assessed. Consistent with data from our in-house samples, relative MBOAT7 mRNA expression was significantly lower in metabolic steatohepatitis patients (Fig. S6F). The hepatic expression of MBOAT7 was also significantly lower in patients harbouring the T- compared to the C-allele (Fig. S6G). Using the same dataset, Volcano plot and Gene Set Enrichment Analysis (GSEA) between the low and high expressed MBOAT7 samples showed overrepresentation of gene in inflammation pathways in MBOAT7 low expressed samples, including arachidonic acid and interferon gamma responses (Fig. S6H, I). Similarly, a negative correlation was found between hepatic MBOAT7 expression and the inflammatory markers (Fig. S6J). In addition, we observed higher sCD163, a macrophage activation marker in patients with MAFLD harbouring the TT genotype and greater hepatic clustering of CD163 and CD68‐positive macrophages, compared to those with the C allele (Fig. S6K). Finally, to address the impact of MBOAT7 on the communication between hepatocytes and macrophages, we examined whether MBOAT7 knockdown in macrophages could increase inflammation in hepatocytes. Co-culture of MBOAT7 knockdown macrophages (MDM and kupffer cells) with hepatocytes increased expression of hepatocyte inflammatory cytokines (Fig. S6L).

### SARS-CoV-2 spike protein S1 subunit downregulates MBOAT7 expression in human macrophages and augments the production of proinflammatory mediators

**Fig. 7:**
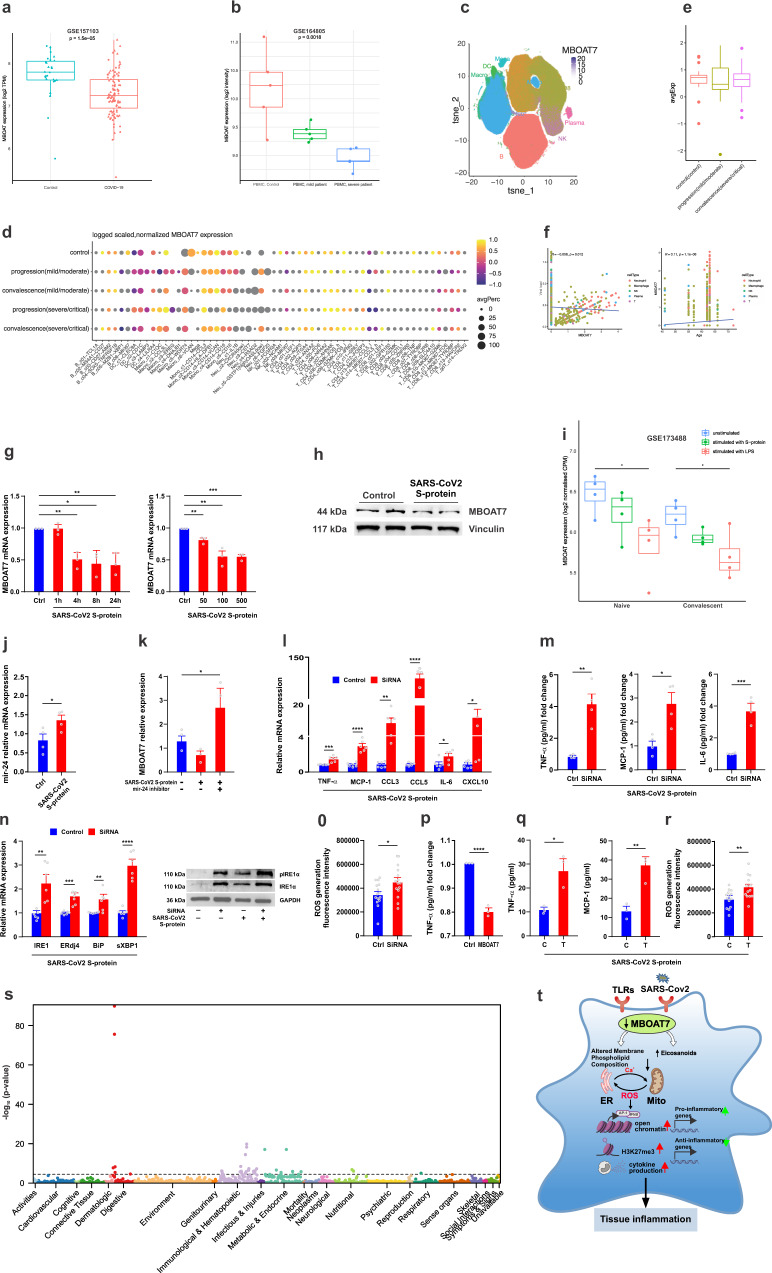
MBOAT7 and COVID-19. **a** Absolute RNA counts of MBOAT7 in leucocytes from blood of patients with COVID-19 and healthy control (GSE157103) (*n* = 126/group), **b** and from PMBCs of mild and severe COVID-19 and healthy controls (GSE164805) (*n* = 15/group). **c** t-SNE plots of 159 PBMC samples scRNA-seq data (GSE158055) coloured by major cell types and expression levels of MBOAT7 in the different types is depicted. NK, natural killer cells; Mono, monocytes; DC, dendritic cells; Mega, megakaryocytes. **d** Dot plots showing the expression of MBOAT7 in cell clusters found in PBMCs. **e** Boxplots of MBOAT7 expression of Mono_c1-CD14-CCL3 clusters from healthy controls (*n* = 20), patients with progression (*n* = 18), and convalescent severe patients (*n* = 35). **f** Pearson’s correlations of MBOAT7 expression with viral load and age and *P*-value are two-sided. **g** SARS-CoV-2 S1 (spike protein) reduces MBOAT7 mRNA expression (*n* = 3) and **h** protein level in MDMs, **i** with a similar trend in the GSE173488 dataset (*n* = 24/group). **j** SARS-CoV-2 S1 upregulates miR-24 expression in MDMs (*n* = 4). **k** miRNA-24 inhibitor reversed the effect of SARS-CoV-2 S1 on MBOAT7 expression levels (*n* = 3). **l** MBOAT7 depletion enhances S1 induced production of pro-inflammatory transcript and **m** protein levels in MDMs, **n** and ER stress markers (*n* = 6; 3 biological replicates and 2 technical replicates in **l** and **n**), **o** and ROS levels (*n* = 15; 3 biological replicates and 5 technical replicates). **p** MBOAT7 activation attenuated inflammatory cytokines levels (*n* = 3). HeLa cells were transfected with full-length 3′UTRs of MBOAT7 containing either the C or the T allele were stimulated by SARS-CoV-2 S1and analyzed for **q** Secreted TNF-α and MCP-1 levels by ELISA (*n* = 3) and **r** ROS generation (*n* = 14; 3 biological replicates and 4–5 technical replicates). **s**) PheWAS of rs8736 with 1498 Phenotypes. Box plots indicate the first and third quartiles with the median in between, the whiskers extend to the furthest point within 1.5× the IQR past the corresponding quartile. *P-*value estimated with two-sided Wilcoxon test. *P < 0.05, ** *P* < 0.01, ****P < 0.001. Source data are provided as a Source Data file. **t** Model of MBOAT7 mechanisms for regulating TLR-induced signalling, cytokines, and ER stress underpinning the increase in COVID-19 severity in patients with MAFLD.

The pathway we have identified—by which MBOAT7 can modulate inflammatory cytokine production in MDMs—would not only be expected to influence the outcome of MAFLD, but also of other diseases involving TLRs dysregulation and these cytokines. COVID-19 severity is driven by severe inflammatory response syndrome (SIRS), with recent data suggesting a crucial role for TLRs^50^. In addition, patients with MAFLD have higher risk for severe COVID-19 and death compared to non-MAFLD patients^51,52^. Thus, to determine whether MBOAT7 plays a role in SARS-CoV-2-induced inflammatory responses, we reanalyzed a publicly available dataset for MBOAT7 expression in immune cells from patients with differing severities of COVID-19. The expression pattern of MBOAT7 was significantly decreased in PMBCs of patients with COVID-19 (Fig. 7a) and this was even more profound among those with severe COVID-19 (Fig. 7b).

To explore changes in MBOAT7 expression in COVID-19, we analyzed a single-cell RNA sequencing (scRNA-seq) dataset of 284 samples from 196 COVID-19 patients; 249 were PBMC samples, each with >1,000 single cells available for the expression levels of MBOAT7 in broad categories of immune cells including neutrophils, macrophages, plasma B cells, T cells, and NK cells^53^. Notable differences in MBOAT7 expression was observed based on the t-distributed stochastic neighbour embedding (t-SNE) projection (Fig. 7c). MBOAT7 expression was significantly downregulated in immune cells from patients with severe COVID-19 compared to healthy patients, particularly in macrophages (Fig. 7d). In addition, MBOAT7 expression was downregulated with the severity of COVID-19 in the Mono_c1-CD14-CCL3 subtype of monocytes (Fig. 7e). This subpopulation expressing large amounts of CCL3 and TNF-α is reported to be highly enriched in patients with severe COVID-19 and likely plays a central role in driving the inflammatory storm^53^. MBOAT7 demonstrated a modest but significant positive correlation with age and a negative correlation with the abundance of viral RNA in the different cell types suggesting an impact of SARS-CoV-2 on MBOAT7 activity (Fig. 7f). To confirm the direct effect of SARS-CoV-2 on MBOAT7 expression in macrophages, we analyzed the effects of S1 protein (spike protein) on MBOAT7 expression in vitro in MDMs. The MBOAT7 transcript was drastically down-regulated after S1 stimulation in a dose and time dependent manner (Fig. 7g) and at protein level (Fig. 7h), consistent with a publicly available dataset (Fig. 7i).

To explore the potential mechanisms, we examined the effect of S1 protein on miR-24 expression in MDMs. The S1 protein (spike protein) upregulated miRNA-24 expression in MDMs (Fig. 6j). To establish the role of miRNA-24 in the S1 protein attenuation of MBOAT7 expression, we conducted complementary studies using an miRNA-24 hairpin inhibitor. This reversed the effect of the S1 protein and increased MBOAT7 expression levels (Fig. 6k). Therefore, changes in miRNA-24 accounts for the observed effect of S1 protein on MBOAT7 expression. However, we realize that additional mechanisms may also be involved.

We then analyzed the effects of MBOAT7 depletion on S1 induced production of pro-inflammatory mediators in human macrophages. As expected, on stimulation with S1 protein, transcript (Fig. 6l) and secreted (Fig. 6m) levels of the pro-inflammatory cytokines were significantly elevated in MBOAT7 depleted cells compared with controls. Similarly, MBOAT7 deficiency enhanced the S1 protein-induced effects on ER stress markers (Fig. 6n) and ROS levels (Fig. 6o). MBOAT7 activation also attenuated inflammatory cytokines levels (Fig. 6p). Next, we investigated whether the *MBOAT7* rs8736 variant regulates these effects. Consistently, the T allele in HeLa cells showed an increase in the levels of pro-inflammatory cytokines (Fig. 6q) and ROS generation (Fig. 6r).

### Human PheWAS analysis identifies an association between MBOAT7 and inflammation-related traits

Finally, based on the importance of MBOAT7 in regulating TLR-driven inflammatory cytokines generation in macrophages, we explored the broader clinical impact of the *MBOAT7* variant. To do this we performed a phenome-wide association (PheWAS) study on the *MBOAT7* SNP rs8736 and 1498 phenotypes from the BioVU biobank. As expected, this unbiased PheWAS identified significant associations of rs8736 with liver injury (e.g., elevated liver enzymes), inflammation and immune-related traits including blood cell traits (myeloid cell count, platelet crit (also called platelet mass) and count, neutrophil count and granulocyte count), and nominally with multiple other traits including TNF-α, periodontitis, appendicitis, and deep vein thrombosis (Fig. 7s and Supplementary Data 1).

## Discussion

We used a multipronged approach to identify MBOAT7 as a repression checkpoint that links the metabolic and epigenetic reprogramming that is required for optimal cellular metabolism, signalling, and transcriptional regulation. MBOAT7 does so by fine-tuning TLR-mediated macrophage effector responses to avoid unchecked amplification of inflammation (Fig. 7t). With the exception of TLR3, which signals exclusively through TRIF, MBOAT7 regulated the signalling of TLR-induced MyD88-dependent pro-inflammatory genes in macrophages. These effects are further regulated by the rs8736 variant in the gene.

We demonstrated that MBOAT7 regulates the metabolic-epigenetic axis to regulate TLR-induced inflammatory responses. As we show, MBOAT7 deficiency mediates a broad range of inflammatory outcomes resulting from eicosanoids generation, oxidative stress, excessive intracellular Ca2^+^, and ROS generation, which are reported to widely affect chromatin structure^54^. Consistently, we showed that MBOAT7 deficiency increased chromatin accessibility and was sufficient to prime cells for enhanced transcriptional responses on TLR stimulation. The resultant reshaping of the epigenetic landscape and transcriptional factor network contributes to a rapid, transcription priming response of “poised” genes upon TLR signalling in the presence of pro-inflammatory cues. In addition, we show that MBOAT7 deficiency selectively exacerbates NLRP3 inflammasome activation but not other inflammasomes. This indicates that MBOAT7 is a gatekeeper of the two activator signals of the NLRP3 inflammasome. Notably, it has been reported that both inhibition of Ca2^+^ mobilization and ROS inhibitors block NLRP3 inflammasome activation but not AIM2 or NLRC4 stimulation^55,56^. These findings suggest an approach that could be employed to selectively target certain actions of the TLRs and inflammasome, potentially allowing manipulation of the immune response in a cell-type restricted fashion.

The effects of MBOAT7 were modulated by the rs8736 variant. We identified that at least one mechanism contributing to lower MBOAT7 expression in rs8736 T allele carriers is a decrease in its transcriptional activity through MBOAT7 mRNA degradation. The latter was mediated by miRNA binding and as we show, is likely attributable to DNA methylation-mediated polyadenylation, and a change in transcript length.

TLRs signalling has been implicated in a wide range of human diseases^57^. In particular, activation of innate immune cells (specially, macrophages) through TLRs is a critical mediator of both COVID‐19 and MAFLD immunopathogenesis^5–7^. We show that MBOAT7 expression in human macrophages is attenuated in both diseases and their severity is influenced by MBOAT7-dependent control of inflammatory responses. Multiple studies have comprehensively demonstrated that MAFLD is a risk factor for progression to severe COVID-19^52,58,59^, but factors that mechanistically drive this association remain elusive. Our data suggest a potential plausible link between fatty liver disease and COVID-19 severity mediated by the interaction of TLRs, MBOAT7, and MBOAT7 genotype. Such a link is further supported by similarities in the range of pro-inflammatory cytokines induced by MBOAT7 repression in patients with severe COVID-19 and in MAFLD, such as TNF-α^60^. Finally, our PheWAS study indicated a broader clinical impact for the pathway we have identified by which MBOAT7 can modulate various inflammatory response and immune-related traits. The dominant effect of MBOAT7 downregulation on liver rather than infectious diseases might be explained by the multiple hits implicated in MAFLD pathogenesis that might be regulated by MBOAT7 including TRL signalling and increases in liver fat content^61,62^. MBOAT7 downregulation was found to increase hepatic fat content by increasing triglyceride synthesis^63^, which causes hepatic inflammation and fibrosis^64^.

In conclusion, our findings define a critical role for MBOAT7 in processes pivotal for systemic immune homoeostasis, including for a broad range of TLR responses. These effects are further regulated by the genotype at *MBOAT7* rs8736. Our findings highlight that modulation of MBOAT7 may provide therapeutic benefit for suppressing inflammation in human diseases associated with dysregulation of the TLR signalling cascade, including in COVID-19 and MAFLD.

## Methods

### Ethics

Ethics approval was obtained from the Sydney West Area Health Service, the University of Sydney, Human Research Ethics Committee (HREC/17/WMEAD/433). Written informed consent was obtained from all participants.

### The reagents

The reagents are described in supplementary Table 2 and primers sequence in Supplementary Table 3.

### Immune cell subsets

Peripheral blood mononuclear cells (PBMCs) were isolated using Ficoll Paque Plus (GE Healthcare). Immune cell isolations were performed using specific kits from StemCell EasySep Kits for the isolation of CD14 as per manufacturer procedures. CD14+ monocytes were cultured at 37 °C and 5% CO_2_ in RPMI medium supplemented with 10% fetal calf serum (FCS) and 50 ng/mL macrophage colony-stimulating factor (M-CSF, Peprotech) or granulocyte macrophage colony-stimulating factor (GM-CSF, Peprotech) for 7 days, replacing media and removing non-adherent cells at day 4.

### Transfection

MBOAT7 plasmid (pCMV6-AC-MBOAT7-GFP, OriGene) and its empty cloning vector pCMV6-ACGFP, OriGene) were transfected into MDMs using a Nucleofector kit (Amaxa) as per manufacturer’s instructions for 48 h. Small interfering RNA (siRNA) transfection was performed with Lipofectamine RNAiMAX (Invitrogen) according to the manufacturer’s instructions using 50 nM of the specific siRNA or non-targeting control for 48 h.

### Treating MDM cells with TLRs, inflammasome agonists, and SARS-CoV-2

Cells were seeded in appropriate plate with 80% confluency and followed by siRNA transfection. 24 h post-cell transfection, cells were treated with Pam3CSK4 (recognized by TLR2) 100 ng/mL, Poly (I: C) (recognized by TLR3) 100 ng/mL, Lipopolysaccharide (LPS) (recognized by TLR4) 500 ng/mL and CpG DNA (recognized by TLR9) 100 ng/mL for the indicated duration of treatment. The concentrations of TLR agonists were according to previous optimization experiments). For COVID-19, cells were treated with SARS-CoV-2 100 ng/ml 4 h. For inflammasome activation, MDMs were incubated with LPS (500 ng/ml, 4 h) and then treated with inflammasome activators as described ATP (1 mM, 30 min), poly(dA:dT) (1 ug/ml, 8 h), MDP (200 ng/ml, 8 h) or flagellin (200 ng/ml, 8 h). Cell supernatants and cell lysates were collected for further analysis.

In another experiment, MDM cells were treated with LPS (500 ng/ml), IFN-γ(20 ng/ml), IL-4 (20 ng/ml), or IL-1β(10 ng/ml) for 24 h and MBOAT7 mRNA expression was assessed by PCR and normalized to untreated MDMs and GAPDH.

### mRNA gene expression

#### RNA Extraction

Total RNA was extracted from cells using the Tissue Total RNA purification Mini Kit (Favorgen Biotech Corp) and Qiagen miRNeasy Mini Kit according to the manufacturer’s instructions. cDNA was reverse transcribed from total RNA using qScript® cDNA SuperMix (Quanta Biosciences, Gaithersburg, MD) according to the manufacturer’s instructions.

#### Real-time quantitative PCR (RT-qPCR)

Real-time PCR was performed on Applied Biosystems, Foster City, CA, USA., using TaqMan Fam labelled expression probes with TaqMan™ Fast Advanced Master Mix or forward and reverse primers for each gene of interest with SYBR Select Master Mix and normalized to the expression of glyceraldehyde 3-phosphate dehydrogenase (GAPDH). Expression was measured using CT values normalized to that of GAPDH (ΔCT = CT (GAPDH) - CT (target) and then expressed as 2^−ΔCT^. Primers sequences and expression probe IDs are provided in the key resource table. For alternative polyadenylation, total and distal isoforms were independently measured and the distal-to-total transcript ratios were calculated.

#### Enzyme-linked immunosorbent assay (ELISA)

Supernatant were assayed for TNF-α, MCP-1, IL-6, and IL-1β levels according to the manufacturer’s instructions using a sandwich enzyme-linked immunosorbent assay (ELISA) (Abcam).

#### Multiplex assay

Supernatant were quantified for 13 human inflammatory cytokines/chemokines, including IL-1β, IFN-α2, IFN-γ, TNF-α, MCP-1, IL-6, IL-8, IL-10, IL-12p70, IL-17A, IL-18, IL-23, and IL-33 using LEGENDplexTM Human Inflammation Panel 1 (13-plex) (Cat. Number: 740809) as per manufacturers’ instructions.

#### Immunofluorescence assay

Cells were rinsed twice with PBS, fixed with ice-cold methanol for 5 min at −20 °C and permeabilized with 0.5 % Triton X-100 for 15 min. Protein block (Dako) was added as blocking solution for 1 h at room temperature. Cells were then incubated with the primary antibody for 1 h at 4 °C. This was followed by incubation with goat anti-rabbit antibodies at 1:1000 dilution in antibody diluent buffer for 1 h at room temperature. The coverslips were placed sample-side down onto ProLong Gold Antifade Mountant with DAPI (Invitrogen, Carlsbad, CA) on the glass slide, then cured for 24 h at room temperature in the dark. The samples were examined using the Olympus FV1000 confocal microscope (60 × magnification). The fluorescence intensity was quantitated using Image J software.

#### Immunohistochemistry

Immunohistochemical staining was done in representative biopsies from seven randomly selected patients with MAFLD with various MBOAT7 genotypes. The primary antibodies were mouse monoclonal anti-human CD163 and anti-human CD68.

#### Western blot analysis

Whole cell extracts were fractionated by SDS-PAGE and transferred to a polyvinylidene difluoride (PVDF) membrane using a transfer apparatus according to the manufacturer’s protocol (Bio-Rad, Hercules, CA). Following transfer, membranes were blocked with 5% non-fat milk in TBST (50 mM Tris, pH 7.4, 150 mM NaCl, 0.05% Tween −20) for 1 h at room temperature. Membranes were washed twice with TBST and incubated with primary antibodies diluted in 5% skim milk/TBST overnight at 4 °C. Following incubation, membranes were washed three times for 10 min with TBST and incubated with secondary antibodies, and diluted in 5% skim milk/TBST for 1 h in the dark at room temperature. Blots were washed with TBST three times and developed with SuperSignal West Pico PLUS Chemiluminescent and SuperSignal West Femto Maximum Sensitivity Substrates (Life Technologies, Australia) according to the manufacturer’s protocol. Membranes were scanned using ChemiDoc Touch Imaging system (Bio-Rad, Hercules, CA).

#### Human mitochondrial DNA (mtDNA) quantification

Mitochondrial DNA was quantified by real-time PCR using the human Mitochondrial DNA (mtDNA) Monitoring Primer Set (Takara) according to the manufacturer’s guidelines. Primer sets for ND1 and ND5 were used for the detection of mtDNA and those of SLCO2B1 and SERPINA1were used for the detection of nuclear DNA.

#### TMRE-mitochondrial membrane potential assay

MDM cells were transfected with MBOAT7 siRNA or scrambled siRNA for 48 h and treated with LPS for 24 h. Mitochondrial Membrane Potential was measured using TMRE dye (Abcam, 113852). Fluorescence intensities were measured immediately on a plate reader at Ex/Em = 549/575 nm. Data were analyzed by subtracting control readings from all measurements and fold change determined from the control.

#### Genotyping

Genotyping for MBOAT7 rs8736 was performed using the genotyping allelic Discrimination method (Applied Biosystems, Foster City, CA) with TaqMan GTXpress ™ Master Mix, catalogue number: 4401857 and TaqMan genotyping probe catalogue number 4351376.

#### Allele-specific expression

To explore whether rs8736‐allele-specific expression contributes to MBOAT7 expression, we quantified both complementary DNA (cDNA) and genomic DNA (gDNA) from 10 subjects heterozygous for the variant using real-time quantitative reverse transcriptase-polymerase chain reaction (RT-PCR).

#### Luciferase assay

The full-length 3’UTR of human MBOAT7 with genetic variation corresponding to C or T of rs8736 was cloned into the pmel/sall restriction site downstream of the gene encoding luciferase in the pmirGLO reporter vector (Promega, Madison, WI). Luciferase activity in cell cultures (human hepatoma cell line (Huh-7) cells (cell bank Australia) and Human embryonic kidney 293 cells (HEK293) cell lines (ATCC)) was assessed using the luciferase assay kit (BPS bioscience) as per manufacturer’s instructions.

#### Measurement of MBOAT7 mRNA stability

To determine MBOAT7 mRNA half-life, Huh-7 cells stably expressing the C or T allele were treated with actinomycin-D (Sigma-Aldrich, St. Louis, MO) at a concentration of 10 μg/mL. RNA samples were then isolated from the cells over a time course of 3 h and subject to qPCR. One-phase decay analysis on GraphPad Prism was performed to determine half-life.

#### Ca2^+^ measurement

Intracellular Ca2^+^ was measured in MDMs using the Fluo-8 Calcium Flux Assay Kit (Abcam, ab112129). In brief, cells were incubated with Fluo-8 for 0.5 h at 37 °C and 0.5 hour at RT in calcium-free Hanks’ balanced salt solution according to the manufacturer’s instructions. Fluorescence was measured in a fluorescent plate reader with an excitation wavelength of 490 nm and emission was measured at 525 nm. Calcium fold was calculated using no stimulation data as the standard value.

#### Enzyme activity assays

Cyclooxygenase (COX), Lipoxygenase Assay, and Cytosolic Phospholipase activity was measured using specific assay Kit (Abcam) according to the manufacturer’s instructions. Caspase-1 activity was stained using FAM-FLICA Caspase-1 Assay kit (ImmunoChemisty Technologies) according to the manufacturer’s instructions.

#### Cholesterol and triglyceride quantification assay

Cholesterol and Triglyceride assay quantification were measured using specific assay kit (Abcam) according to the manufacturer’s instructions.

#### XF cell mito stress assay

XF Cell Mito Stress test was used to measure key parameters of mitochondrial function by directly measuring the oxygen consumption rate (OCR) and extracellular acidification rate (ECAR). OCR and ECAR were examined with the XFe24 Analyzer (Seahorse Bioscience, Agilent Technologies). As described by the manufacturer’s protocol, cells were seeded into the XF24 cell culture microplate (Seahorse Bioscience) at a density of 200,000 cells per well. After overnight culture, cells were transfected with MBOAT7 siRNA or scramble. 48 h-post transfection, cells were left untreated or treated with LPS for 3 h. Cells were then washed and replaced with Seahorse XF RPMI media supplemented with 1 mM pyruvate, 2 mM glutamine and 10 mM glucose, pH 7.4. Following incubation in an incubator without CO_2_ at 37 °C for 60 min, the Mito stress assay was processed by sequential addition of 2 µM oligomycin, 2 µM FCCP, and 0.5 µM rotenone/antimycin A. XFe Wave software (Seahorse Bioscience) was used to analyze the results.

#### Cellular reactive oxygen species (ROS) assay

MDM cells were transfected with MBOAT7 siRNA or scrambled siRNA for 48 h and treated with LPS for 24 h. Intracellular ROS was measured using the redox-sensitive dye DCFDA/H2DCFDA- cellular ROS Assay Kit (Abcam, ab113851). Fluorescence intensities were measured immediately on a plate reader at Ex/Em = 485/535 nm. Red fluorescent dye MitoSOX (MitoSOX™ Red Mitochondrial Superoxide Indicator) was used to measure mitochondrial ROS. Data were analyzed by subtracting control readings from all measurements and fold change determined from the control.

#### Electron microscopy

Human MDM cells were incubated in Karnovsky’s fixative for 4 h, washed in 0.1 M phosphate buffer, and resuspended in 10% bovine serum albumin (BSA fraction V) for 20 min. Excess BSA was removed and Karnovsky’s fixative layered on top to form a solid encapsulated pellet at 4 °C overnight. Trimmed blocks were post fixed in 2% osmium tetroxide in 0.1 M cacodylate buffer for 2 h, rinsed in water, then dehydrated in an ascending series of ethanol before transferring to acetone. Cells were infiltrated with a mixture of acetone/resin (TAAB TLV soft resin) followed by several changes of pure resin. Samples were embedded and cured overnight at 70 °C. Sections were cut at 90 nm using a Leica UC6 ultramicrotome (Leica Microsystems) and stained with 2% uranyl acetate in 50% ethanol (10 min) and Reynold’s lead citrate (4 min). Grids were examined with a Jeol 1400 Plus Transmission Electron Microscope operating at 80 kV. Images were collected using a digital sCMOS “Flash” camera with the “Limitless Panorama” wide area auto montage software. Morphometric measurements of the mitochondrial area, perimeter and length were completed using the free hand tool in the Jeol SightX Image Viewer software by manually tracing around the mitochondria.

#### Phospholipids analysis

Phospholipids were measured in MDM samples from 6 participants as previously described in detail^65^ (The Mass Spectrometry Lipidomics Core Facility, University of Colorado Anschutz Medical Campus, Aurora, Colorado). Briefly, all conditions are based on the same number of cells (1 × 10^6^). After addition of the deuterated internal standards (25 ng each), samples were extracted according to the method of Bligh and Dyer^66^. Samples were injected into an HPLC system connected to a triple quadrupole mass spectrometer (API3200, AB SCIEX, Framingham, MA) and normal-phase chromatography was performed using a silica HPLC column (Ascentis, 150 × 2.1 mm, 5 μm, Supelco, Bellefonte, PA) at a flow rate of 200 μL/min. Mass spectrometric analyses were performed in the negative ion mode using multiple reaction monitoring (MRM) for specific analytes, plus the deuterated standards. Results are reported as the ratio between the integrated area of each analyte and the integrated area of the corresponding internal standard for each class and data are provided in Supplementary Data 2. Metabolites were discriminated using orthogonal projections to latent structure discriminant analysis (OPLS-DA). The class prediction model was validated by three-fold cross-validation repeated 50 times. A potential limitation is that no adjustment for multiple testing was made as this was an exploratory study.

#### Eicosanoids analysis

Eicosanoids were measured in MDM samples from 5 to 6 participants at a concentration of 1 × 10^6^ cells/mL, as previously described in detail^65^ (The Mass Spectrometry Lipidomics Core Facility, University of Colorado Anschutz Medical Campus, Aurora, Colorado). Briefly, after the addition of the internal standards(10 ng), an Ultra triple quadrupole mass spectrometer equipped with an ESI ion source (Thermo Fisher Scientific) was used. All compounds were analyzed in a negative ion polarity mode. Eicosanoids were quantified by multiple reaction monitoring (MRM). The MRM transitions were monitored as previously described^65^

### RNA-seq

#### RNA-seq library generation

Total RNA was isolated from MDMs transfected with MBOAT7 siRNA or control with and without LPS using the RNA mini kit (Qiagen). RNA purity and integrity were confirmed using an Agilent Bioanalyzer. Sequencing libraries were prepared from 100–500 ng of total RNA using the TrueSeq RNA sample preparation kit v2 (Illumina). Briefly, mRNA was purified, fragmented, and used for first- and second-strand cDNA synthesis followed by adenylation of 3′-ends. Samples were ligated to unique adaptors and subjected to PCR amplification. Libraries were validated using the 2100 BioAnalyzer (Agilent), normalized and pooled for sequencing. RNA-seq libraries prepared from three biological replicates for each condition were sequenced on the Illumina HiSeq 2000 using barcoded multiplexing and a 100 bp read length. Five biological replicates were performed.

#### High-throughput sequencing and analysis

Single-end 100 bp RNA-sequencing was performed on an Illumina platform by the Australian Genome Research Facility (AGRF), Melbourne, Australia. This yielded a median of 31 M usable reads per sample. Read alignment and junction mapping to genome build GRCh37 were accomplished using STAR version 2.7.6a^67^. Known splice junctions from the University of California Santa Cruz (UCSC) hg19 genome annotation were supplied to the aligner and de novo junction discovery was also permitted. Differential gene expression analysis and statistical testing were performed using Cuffdiff2 version 2.2.1^68^, employing the hg19 genome annotation. Transcript expression was calculated as gene-level relative abundance in fragments per kilobase of exon model per million mapped fragments and employed correction for transcript abundance bias. RNA-seq results for genes of interest were also explored visually using the UCSC Genome Browser. The software tool Ingenuity Pathway Analysis (IPA) was used for pathway discovery and gene ontology enrichment.

#### ATAC-seq

In the assay, intact nuclei are treated with a hyperactive Tn5 transposase mutant which is able to simultaneously tag the target DNA with sequencing adapters to fragment the DNA in a process termed “tagmentation”. The ATAC-Seq Kit (Catalogue No. 53150 Active Motif North America) was used for tagmentation and library preparation as per kit procedures. Briefly, cells were counted and aliquots of 50,000 to 100,000 cells were centrifuged at 500 × *g* for 5 min at 4 °C. Then the pellet was resuspended in 100 μl ice-cold ATAC Lysis Buffer. 50 μl of Tagmentation Master Mix was added to each sample and incubated at 37 °C for 30 min in a thermomixer set at 800 rpm. This was followed by extraction and amplification of the tagmented DNA with library preparation using unique combinations of i7/i5 primers which are based on Illumina’s Nextera adapters. Finally, the library was purified using SPRI bead clean-up. Two biological replicates were performed.

#### Cut&Tag

Cleavage Under Targets and Tagmentation (CUT&Tag) is a method to investigate genomic localization of histone modifications and some transcription factors to reveal interactions between proteins and DNA or identifying DNA binding sites for proteins of interest. CUT&Tag is based on the same principles as ChIP-Seq but with several changes to the protocol, where fresh cells are bound to concanavalin A beads and the antibody incubation is performed with cells in their native state. Directly following antibody binding, the chromatin is sheared, tagmentation performed using the protein A-Tn5 (pA-Tn5) transposome and NGS libraries are prepared. CUT&Tag-IT™ Assay Kits (Catalogue No. 53160Active Motif North America) were used for tagmentation and library preparation as per kit procedures. Briefly, 50,000 to 100,000 cells were harvested and washed with 1× wash Buffer supplemented with Protease Inhibitor Cocktail. Then the cells were bound to Concanavalin A beads and incubated with 1ry antibody overnight at 4 °C with orbital mixing. This was followed by 2 h incubation with Guinea Pig Anti-Rabbit Antibody secondary antibody 1:100 in Dig-Wash Buffer. Cells were incubated for 60 min with pA-Tn5 Transposomes diluted to 1:100 in Dig-300 buffer. Then 50 μl of Tagmentation Master Mix was added to each sample and incubated at 37 °C for 30 min in a thermomixer set at 800 rpm. This was followed by extraction and amplification of tagmented DNA with library preparation using unique combinations of i7/i5 primers which are based on Illumina’s Nextera adapters. Finally, the library was purified by SPRI bead clean-up. Two biological replicates were performed.

#### High-throughput ATAC and cut & tag sequencing analysis

Paired-end 100 bp sequencing was performed on an Illumina platform by the AGRF, yielding 30–40 M paired reads per sample. Both the ATAC-seq and Cut & Tag utilize a Nextera transposase sequence. As expected for this method, approximately 25% of reads exhibited 3’ contamination by this sequence, which was removed prior to alignment using Trimmomatic version 0.39^69^. Read alignment was performed using Bowtie2 version 2.4.4^70^ in end-to-end and very sensitive modes using the GRCh37 reference sequence. Peak finding was accomplished using Homer version 4.11 (http://homer.ucsd.edu/homer/) using the following settings: ATAC-seq; -region -size 300-minDist 1000-fdr 0.0001, Cut & Tag; -region -size 1000 -minDist 2500 -fdr 0.0001. Differential peak analysis between samples was performed using Homer programme getDifferentialPeaks.

#### DNA methylation

Measurement of region-specific DNA methylation was done by the OneStep qMethyl™ Kit (catalogue No. D5310 Zymo research) as per manufacturer’s instructions. Briefly, measurement of region-specific DNA methylation was done by selective amplification of methylated cytosines in the CpG dinucleotide context. This is accomplished by splitting any DNA to be tested into two parts: a “Test Reaction” and a “Reference Reaction”. DNA in the Test Reaction is digested with Methylation Sensitive Restriction Enzymes (MSREs) while DNA in the Reference Reaction is not. The DNA from both samples is then amplified using real-time PCR in the presence of SYTO® 9 fluorescent dye and quantified. Cycle threshold (Ct) values for Test and Reference DNA samples will vary depending on methylation status, with large Ct differences most characteristic of non-methylated DNA.

#### Phenome-wide association study

A Phenome-wide association studies (PheWAS) for rs8736 was conducted in the available GWAS summary statistics on the Complex Traits Genetics Virtual Lab (CTG-VL), pheweb and GWAS-ATLAS separately^71,72^, resulting in 1498 phenotypes (Table S2). Most of these phenotypes are sourced from the UK Biobank. SNP (rs8736) to phenotype association was tested correcting for multiple testing through the Bonferroni correction (*P*-value = 3.33E−05).

#### Data analysis and statistics

Statistical analysis was performed using GraphPad Prism software version 7.0 (GraphPad Software Inc., San Diego, CA) and expressed as mean ± standard error of the mean (SEM). One-way analysis of variance was performed for multiple group analyses whereas the student’s t-test was performed when comparing two groups.

Expression analyses were performed in the R Statistical Environment 58 with tidyverse 59. scRNA MBOAT7 data were accessed for GSE158055, normalized to 1e4 and scaled. Cells with scaled expression of >0 were considered positive and values were logged for calculation and plotting. Normalized data for expression arrays (GSE164805) and RNAseq (GSE171110,GSE173488,GSE160351, and GSE157103) were accessed from GEO using GEOquery^73^ and log2 transformed before comparing MBOAT7 to sample annotations. For differential gene expression (DGE) GSE157103 counts were accessed for COVID samples and then binned according to their MBOAT7 expression. Peak motif enrichment was performed using the findMotifs.pl tool in Homer and read enrichment +/−2kbp around peaks and ENCODE v2 chipseq peaks for transcription factors was plotted using deeptools^74^. A significant difference was assumed for *p* values (**** *p* ≤ 0.0001, *** *p* ≤ 0.001, **p ≤ 0.01, * *p* ≤ 0.05).

### Reporting summary

Further information on research design is available in the Nature Portfolio Reporting Summary linked to this article.

## Supplementary information

Supplementary Information

Description of Additional Supplementary Files

Supplementary Data 1

Supplementary Data 2

Reporting Summary

## Data Availability

RNA-Seq, ATAC-seq and Cut&Tag datasets have been deposited at NCBI GEO dataset and are publicly available under the accession number: GSE204965. The rest of the data are available within the main text and supplementary file. Source data are provided with this paper. This paper analyses existing publicly available data. These datasets’ accession numbers are (GSE158055, GSE164805, GSE171110, GSE173488, GSE160351, GSE157103 and E-MEXP-3291) Source data are provided with this paper.

## Source data

Source Data

## References

[1] Alharthi J, Latchoumanin O, George J, Eslam M (2020). Macrophages in metabolic associated fatty liver disease. World J. Gastroenterol..

[2] O’Neill LAJ (2008). When signaling pathways collide: positive and negative regulation of Toll-like receptor signal transduction. Immunity.

[3] Fujihara M (2003). Molecular mechanisms of macrophage activation and deactivation by lipopolysaccharide: roles of the receptor complex. Pharm. Ther..

[4] Biswas SK, Mantovani A (2012). Orchestration of metabolism by macrophages. Cell Metab..

[5] Miura K (2013). Toll-like receptor 2 and palmitic acid cooperatively contribute to the development of nonalcoholic steatohepatitis through inflammasome activation in mice. Hepatology.

[6] Kawai T, Akira S (2007). TLR signaling. Semin. Immunol..

[7] Jialal I, Kaur H, Devaraj S (2014). Toll-like receptor status in obesity and metabolic syndrome: a translational perspective. J. Clin. Endocrinol. Metab..

[8] Thabet K (2016). MBOAT7 rs641738 increases risk of liver inflammation and transition to fibrosis in chronic hepatitis C. Nat. Commun..

[9] Thabet K (2017). The membrane-bound O-acyltransferase domain-containing 7 variant rs641738 increases inflammation and fibrosis in chronic hepatitis B. Hepatology.

[10] Eslam M, George J (2019). Genetic insights for drug development in NAFLD. Trends Pharmacol. Sci..

[11] Mancina RM (2016). The MBOAT7-TMC4 variant rs641738 increases risk of nonalcoholic fatty liver disease in individuals of European descent. Gastroenterology.

[12] Machill, A., Bals, R., Lammert, F. & Krawczyk, M. Genetic insight into COVID‐19 related liver injury: a note on MBOAT7. In *Liver International* (2020). 10.1111/liv.14732 PMC7753702 33202092

[13] Caddeo A (2019). MBOAT7 is anchored to endomembranes by six transmembrane domains. J. Struct. Biol..

[14] Gijon MA, Riekhof WR, Zarini S, Murphy RC, Voelker DR (2008). Lysophospholipid acyltransferases and arachidonate recycling in human neutrophils. J. Biol. Chem..

[15] Mejia EM, Hatch GM (2016). Mitochondrial phospholipids: role in mitochondrial function. J. Bioenerg. Biomembr..

[16] Fagone P, Jackowski S (2009). Membrane phospholipid synthesis and endoplasmic reticulum function. J. Lipid Res..

[17] West AP (2011). TLR signalling augments macrophage bactericidal activity through mitochondrial ROS. Nature.

[18] Zhou R, Yazdi AS, Menu P, Tschopp J (2011). A role for mitochondria in NLRP3 inflammasome activation. Nature.

[19] Miura K (2016). Toll-like receptor 4 on macrophage promotes the development of steatohepatitis-related hepatocellular carcinoma in mice. J. Biol. Chem..

[20] Sharifnia T (2015). Hepatic TLR4 signaling in obese NAFLD. Am. J. Physiol.-Gastrointest. Liver Physiol..

[21] Luci, C., Bourinet, M., Leclère, P. S., Anty, R. & Gual, P. Chronic inflammation in non-alcoholic steatohepatitis: molecular mechanisms and therapeutic strategies. *Front. Endocrinol.* **11**, 597648 (2020). 10.3389/fendo.2020.597648 PMC7771356 33384662

[22] Kawai, T. & Akira, S. *Seminars in Immunology* 19 24–32 (Elsevier, 2007). 10.1016/j.smim.2006.12.004 17275323

[23] Nanavaty V (2020). DNA methylation regulates alternative polyadenylation via CTCF and the cohesin complex. Mol. Cell.

[24] Rastogi P, McHowat J (2009). Inhibition of calcium-independent phospholipase A2 prevents inflammatory mediator production in pulmonary microvascular endothelium. Respir. Physiol. Neurobiol..

[25] Chu J (2015). Pharmacologic blockade of 12/15-lipoxygenase ameliorates memory deficits, A beta and tau neuropathology in the triple-transgenic mice. Mol. Psychiatry.

[26] Shirey KA (2014). Role of the lipoxygenase pathway in RSV-induced alternatively activated macrophages leading to resolution of lung pathology. Mucosal Immunol..

[27] Maiers, J. L. & Malhi, H. *Seminars in Liver Disease* 39 235 (NIH Public Access, 2019). 10.1055/s-0039-1681032 PMC6530577 30912096

[28] Grootjans J, Kaser A, Kaufman RJ, Blumberg RS (2016). The unfolded protein response in immunity and inflammation. Nat. Rev. Immunol..

[29] Cross BC (2012). The molecular basis for selective inhibition of unconventional mRNA splicing by an IRE1-binding small molecule. Proc. Natl Acad. Sci. USA.

[30] Luo X, He Q, Huang Y, Sheikh M (2005). Transcriptional upregulation of PUMA modulates endoplasmic reticulum calcium pool depletion-induced apoptosis via Bax activation. Cell Death Differ..

[31] Yaron J (2015). K+ regulates Ca2+ to drive inflammasome signaling: dynamic visualization of ion flux in live cells. Cell Death Dis..

[32] Bronner DN (2015). Endoplasmic reticulum stress activates the inflammasome via NLRP3- and caspase-2-driven mitochondrial damage. Immunity.

[33] Weinberg SE, Sena LA, Chandel NS (2015). Mitochondria in the regulation of innate and adaptive immunity. Immunity.

[34] Tur J, Vico T, Lloberas J, Zorzano A, Celada A (2017). Macrophages and mitochondria: a critical interplay between metabolism, signaling, and the functional activity. Adv. Immunol..

[35] Boveris A, Cadenas E (1997). Cellular sources and steady-state levels of reactive oxygen species. Lung Biol. Health Dis..

[36] Lee HM (2013). Upregulated NLRP3 inflammasome activation in patients with type 2 diabetes. Diabetes.

[37] Rambold AS, Pearce EL (2018). Mitochondrial dynamics at the interface of immune cell metabolism and function. Trends Immunol..

[38] Brookes PS (2005). Mitochondrial H+ leak and ROS generation: an odd couple. Free Radic. Biol. Med..

[39] Hüttemann M (2008). Regulation of oxidative phosphorylation, the mitochondrial membrane potential, and their role in human disease. J. Bioenerg. Biomembranes.

[40] Zhang Y (2016). Mitoguardin regulates mitochondrial fusion through MitoPLD and is required for neuronal homeostasis. Mol. Cell.

[41] Archer SL (2013). Mitochondrial dynamics—mitochondrial fission and fusion in human diseases. N. Engl. J. Med..

[42] Choi AJ, Ryter SW (2014). Inflammasomes: molecular regulation and implications for metabolic and cognitive diseases. Molecules Cells.

[43] Ivashkiv LB (2013). Epigenetic regulation of macrophage polarization and function. Trends Immunol..

[44] Lawrence T, Natoli G (2011). Transcriptional regulation of macrophage polarization: enabling diversity with identity. Nat. Rev. Immunol..

[45] Kreuz S, Fischle W (2016). Oxidative stress signaling to chromatin in health and disease. Epigenomics.

[46] Swigut T, Wysocka J (2007). H3K27 demethylases, at long last. Cell.

[47] Zhang X (2018). Macrophage/microglial Ezh2 facilitates autoimmune inflammation through inhibition of Socs3. J. Exp. Med..

[48] Negishi H (2005). Negative regulation of Toll-like-receptor signaling by IRF-4. Proc. Natl Acad. Sci. USA.

[49] Li P (2012). BATF–JUN is critical for IRF4-mediated transcription in T cells. Nature.

[50] Schultze, J. L. & Aschenbrenner, A. C. COVID-19 and the human innate immune system. *Cell* **184**, 1671–1692 (2021). 10.1016/j.cell.2021.02.029 PMC7885626 33743212

[51] Targher G (2020). Risk of severe illness from COVID-19 in patients with metabolic dysfunction-associated fatty liver disease and increased fibrosis scores. Gut.

[52] Zhou YJ (2020). Metabolic-associated fatty liver disease is associated with severity of COVID-19. Liver Int..

[53] Ren X (2021). COVID-19 immune features revealed by a large-scale single-cell transcriptome atlas. Cell.

[54] Zheng, M. et al. TLR2 senses the SARS-CoV-2 envelope protein to produce inflammatory cytokines. *Nat. Immunol.* **22**, 1–10 (2021). 10.1038/s41590-021-00937-x PMC8882317 33963333

[55] Bauernfeind F (2011). Cutting edge: reactive oxygen species inhibitors block priming, but not activation, of the NLRP3 inflammasome. J. Immunol..

[56] Murakami T (2012). Critical role for calcium mobilization in activation of the NLRP3 inflammasome. Proc. Natl Acad. Sci. USA.

[57] Lin Y-T, Verma A, Hodgkinson CP (2012). Toll-like receptors and human disease: lessons from single nucleotide polymorphisms. Curr. Genom..

[58] Zhou Y-J (2020). Younger patients with MAFLD are at increased risk of severe COVID-19 illness: a multicenter preliminary analysis. J. Hepatol..

[59] Ji D (2020). Non-alcoholic fatty liver diseases in patients with COVID-19: a retrospective study. J. Hepatol..

[60] Giamarellos-Bourboulis EJ (2020). Complex immune dysregulation in COVID-19 patients with severe respiratory failure. Cell Host Microbe.

[61] Bayoumi A, Grønbæk H, George J, Eslam M (2020). The epigenetic drug discovery landscape for metabolic-associated fatty liver disease. Trends Genet..

[62] Eslam M (2020). MAFLD: a consensus-driven proposed nomenclature for metabolic associated fatty liver disease. Gastroenterology.

[63] Tanaka Y (2021). LPIAT1/MBOAT7 depletion increases triglyceride synthesis fueled by high phosphatidylinositol turnover. Gut.

[64] Dongiovanni P (2018). Causal relationship of hepatic fat with liver damage and insulin resistance in nonalcoholic fatty liver. J. Intern. Med..

[65] Zarini S, Hankin JA, Murphy RC, Gijón MA (2014). Lysophospholipid acyltransferases and eicosanoid biosynthesis in zebrafish myeloid cells. Prostaglandins Other Lipid Mediat..

[66] Bligh EG, Dyer WJ (1959). A rapid method of total lipid extraction and purification. Can. J. Biochem. Physiol..

[67] Dobin A (2013). STAR: ultrafast universal RNA-seq aligner. Bioinformatics.

[68] Trapnell C (2013). Differential analysis of gene regulation at transcript resolution with RNA-seq. Nat. Biotechnol..

[69] Bolger AM, Lohse M, Usadel B (2014). Trimmomatic: a flexible trimmer for Illumina sequence data. Bioinformatics.

[70] Langmead B, Salzberg SL (2012). Fast gapped-read alignment with Bowtie 2. Nat. Methods.

[71] Taliun SAG (2020). Exploring and visualizing large-scale genetic associations by using PheWeb. Nat. Genet..

[72] Watanabe K (2019). A global overview of pleiotropy and genetic architecture in complex traits. Nat. Genet..

[73] Davis S, Meltzer PS (2007). GEOquery: a bridge between the Gene Expression Omnibus (GEO) and BioConductor. Bioinformatics.

[74] Ramírez F (2016). deepTools2: a next generation web server for deep-sequencing data analysis. Nucleic Acids Res..

