# Supplementary material for: A metabolic associated fatty liver disease risk variant in MBOAT7 regulates toll like receptor induced outcomes
Source: Nat Commun. 2022 Dec 6;13:7430. doi: 10.1038/s41467-022-35158-9 (PMC9726889; doi:10.1038/s41467-022-35158-9)
Supplement: Supplementary file 1 — Supplementary Information [file 41467_2022_35158_MOESM1_ESM.pdf]

A metabolic associated fatty liver disease risk variant in MBOAT7 regulates toll like receptor induced outcomes

Jawaher Alharthi, Ali Bayoumi, Khaled Thabet, Ziyan Pan, Brian S. Gloss, Olivier Latchoumanin, Mischa Lundberg, Natalie A. Twine, Duncan McLeod, Shafi Alenizi, Leon A. Adams, Martin Weltman, Thomas Berg, Christopher Liddle, Jacob George, Mohammed Eslam.

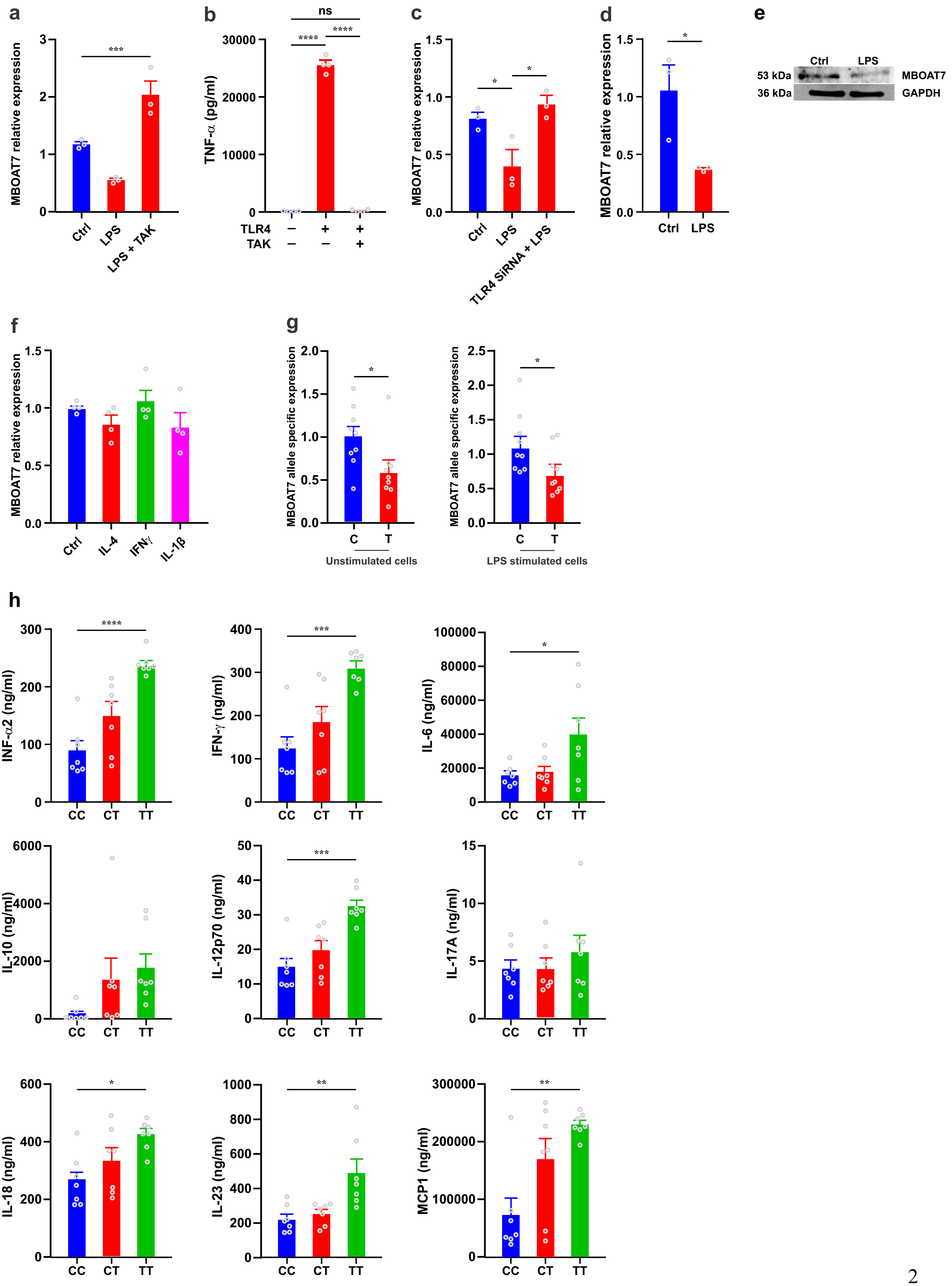

a
MBOAT7 relative expression
***
Ctrl
LPS
LPS + TAK
b
TNF-α (pg/ml)
ns
****
****
TLR4
TAK
c
MBOAT7 relative expression
*
*
Ctrl
LPS
TLR4 SiRNA + LPS
d
MBOAT7 relative expression
*
Ctrl
LPS
e
Ctrl
LPS
53 kDa
36 kDa
MBOAT7
GAPDH
f
MBOAT7 relative expression
Ctrl
IL-4
IFNγ
IL-1β
g
MBOAT7 allele specific expression
*
C
T
Unstimulated cells
LPS stimulated cells
h
INF-α2 (ng/ml)
IFN-γ (ng/ml)
IL-6 (ng/ml)
IL-10 (ng/ml)
IL-12p70 (ng/ml)
IL-17A (ng/ml)
IL-18 (ng/ml)
IL-23 (ng/ml)
MCP1 (ng/ml)
CC
CT
TT
****
***
**

Supplementary Figure 1: a) Human MDMs from subjects heterozygous for rs8736 (n = 3) were stimulated with lipopolysaccharide (LPS) (TLR4) and / or a specific TLR4 inhibitor (TAK-242) for 24 hours and mRNA expression of MBOAT7 was analyzed by PCR and normalized to GAPDH in response to LPS stimulation. **b)** TNF-α levels in conditioned media measured by ELISA. **c)** Human MDMs from subjects heterozygous for rs8736 (n = 3) were stimulated with lipopolysaccharide (LPS) (TLR4) and / or a specific TLR4 SiRNA for 24 hours and mRNA expression of MBOAT7 was analyzed by PCR and normalized to GAPDH in response to LPS stimulation. **d)** Human hepatic Kupffer cells were treated with LPS for 24 hours and then examined for MBOAT7 relative mRNA expression by PCR and normalized to GAPDH, **e)** MBOAT7 protein expression by Western blot in MDMs. **f**) Human MDMs from subjects heterozygous for rs8736 were stimulated with IFN-γ, IL-4 or IL-1β for 24 hours and MBOAT7 mRNA expression was assessed by PCR and normalized to untreated MDMs and GAPDH. **g**) Allele-specific expression assay showing the ratio of rs8736 alleles in genomic DNA (gDNA) and complementary DNA (cDNA; synthesized from pre-mRNA) from MDMs of heterozygous individuals either unstimulated stimulated with LPS for 24 hours (n = 9). **h**) IFN-α2, IFN-γ, IL-6, IL-10, IL-12p70, IL-17A, IL-18, IL-23, and MCP-1 production by MDMs stratified by rs8736 genotype following 24 hours stimulation with Pam3Cys4 [TLR1/2] (n = 7 per genotype). P-value < 0.04 for all comparisons.Values are represented by vertical bars and are mean ± sem; *P < 0.05, ** P < 0.01, ****P < 0.001. Statistical differences between groups were assessed by 2-tailed Student's t test or one-way ANOVA; multiple comparisons were corrected by Bonferroni correction. Source data are provided as a Source Data file.

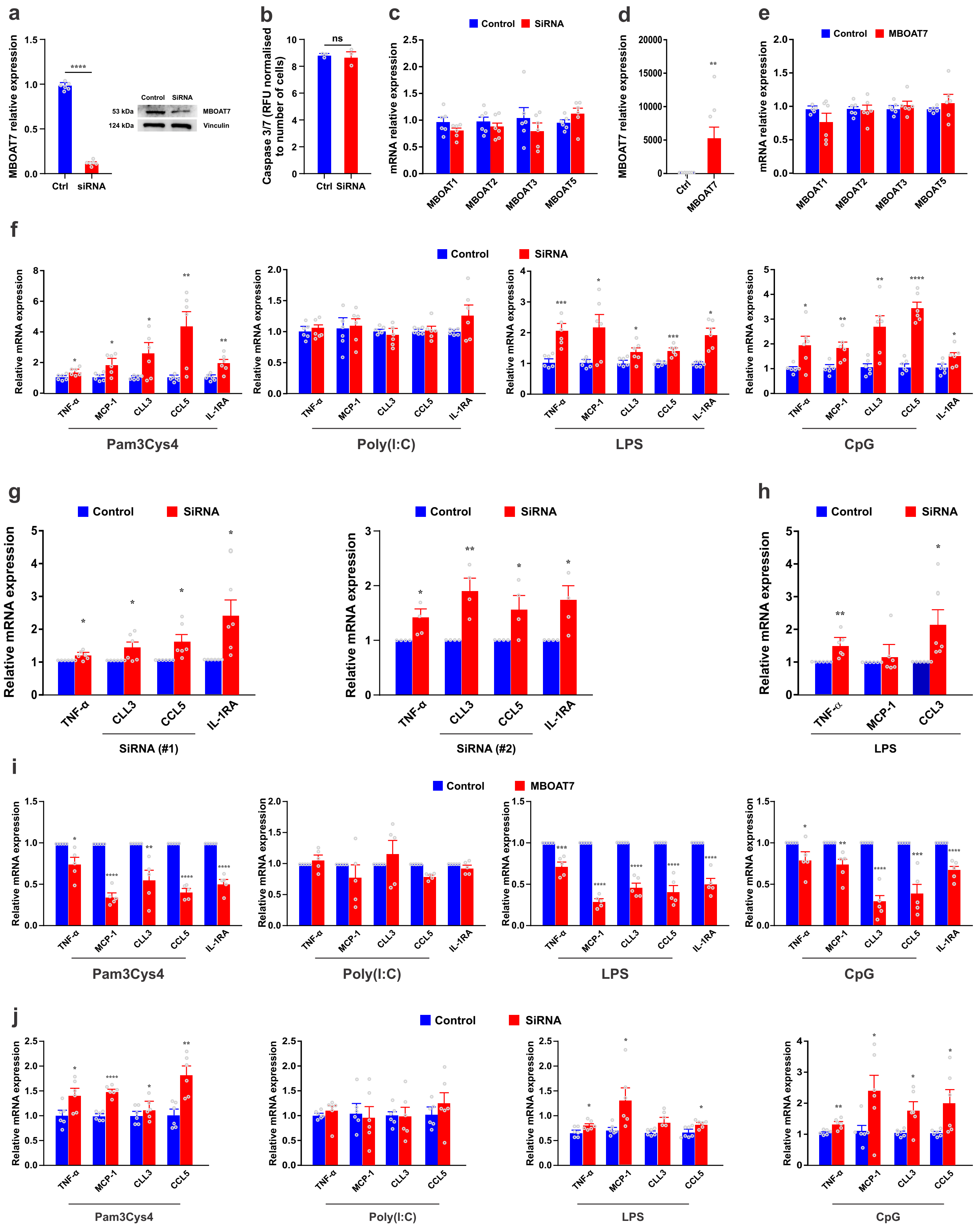
a
MBOAT7 relative expression
Ctrl
siRNA
Control
SiRNA
53 kDa
124 kDa
MBOAT7
Vinculin
b
Caspase 3/7 (RFU normalised to number of cells)
ns
Ctrl
SiRNA
c
Control
SiRNA
mRNA relative expression
MBOAT1
MBOAT2
MBOAT3
MBOAT5
d
MBOAT7 relative expression
Ctrl
MBOAT7
e
Control
MBOAT7
mRNA relative expression
f
Control
SiRNA
Relative mRNA expression
TNF-α
MCP-1
CLL3
CCL5
IL-1RA
Pam3Cys4
Poly(I:C)
LPS
CpG
g
Control
SiRNA
Relative mRNA expression
CLL3
SiRNA (#1)
SiRNA (#2)
h
Control
SiRNA
Relative mRNA expression
LPS
i
Control
MBOAT7
Relative mRNA expression
Pam3Cys4
Poly(I:C)
LPS
CpG
j
Control
SiRNA
Relative mRNA expression
Pam3Cys4
Poly(I:C)
LPS
CpG

Supplementary Figure 2: a) Human MDMs were transfected with scrambled or MBOAT7 siRNA for 48 hours and MBOAT7 relative mRNA expression was assessed by RT-PCR and normalized to GAPDH and MBOAT7 protein by Western normalised to α-tubulin. **b)** Transfected cells were assessed for cell viability using Caspase 3/7 activity; shown is RFU normalised to number of cells (n=3). **c)** The expression of the MBOAT family were determined by RT-PCR and normalized to GAPDH. Human MDMs were transfected with control empty vector or an MBOAT7 vector for 48 hours and **d)** MBOAT7 relative mRNA expression was assessed by RT-PCR (n=4). **e)** The expression of MBOAT family members were determined by RT-PCR. **f)** Human MDMs were transfected with MBOAT7 siRNA or scramble as control and treated with the indicated TLRs ligands (Pam3Cys4 [TLR1/2], Poly(I:C) [TLR3], LPS [TLR4] and CpG [TLR9]) for 24 hours. Shown is relative mRNA expression of different cytokines assessed by RT-PCR and normalized to GAPDH. **g)** Relative mRNA expression of different cytokines in MDMs transfected with 2 additional MBOAT7 siRNAs or scramble as control and treated with LPS for 24 hours. **h)** Human Kupffer cells were transfected with MBOAT7 siRNA or scramble as control and treated with LPS for 24 hours. Shown is relative mRNA expression of different cytokines by RT-PCR. **i)** MDMs were transfected with MBOAT7 overexpression plasmid or the empty vector as control and treated with the indicated TLRs ligands for 24 hours. Shown is relative mRNA expression of different cytokines assessed by RT-PCR and normalized to GAPDH (n=3). **j)** Human MDMs were cultured with GM-CSF and transfected with MBOAT7 siRNA or scramble and treated with the indicated TLR ligands for 24 hours. Shown is relative mRNA expression of different cytokines assessed by RT-PCR and normalized to GAPDH. (n = 6 includes 3 biological replicates and 2 technical replicates in **a, c, e, f, g, h** and **j**). P-value < 0.04 for all comparisons. Values are represented by vertical bars and are mean ± sem; *P < 0.05, ** P < 0.01, ****P < 0.001. Statistical differences between groups were assessed by 2-tailed Student's t test. Source data are provided as a Source Data file.

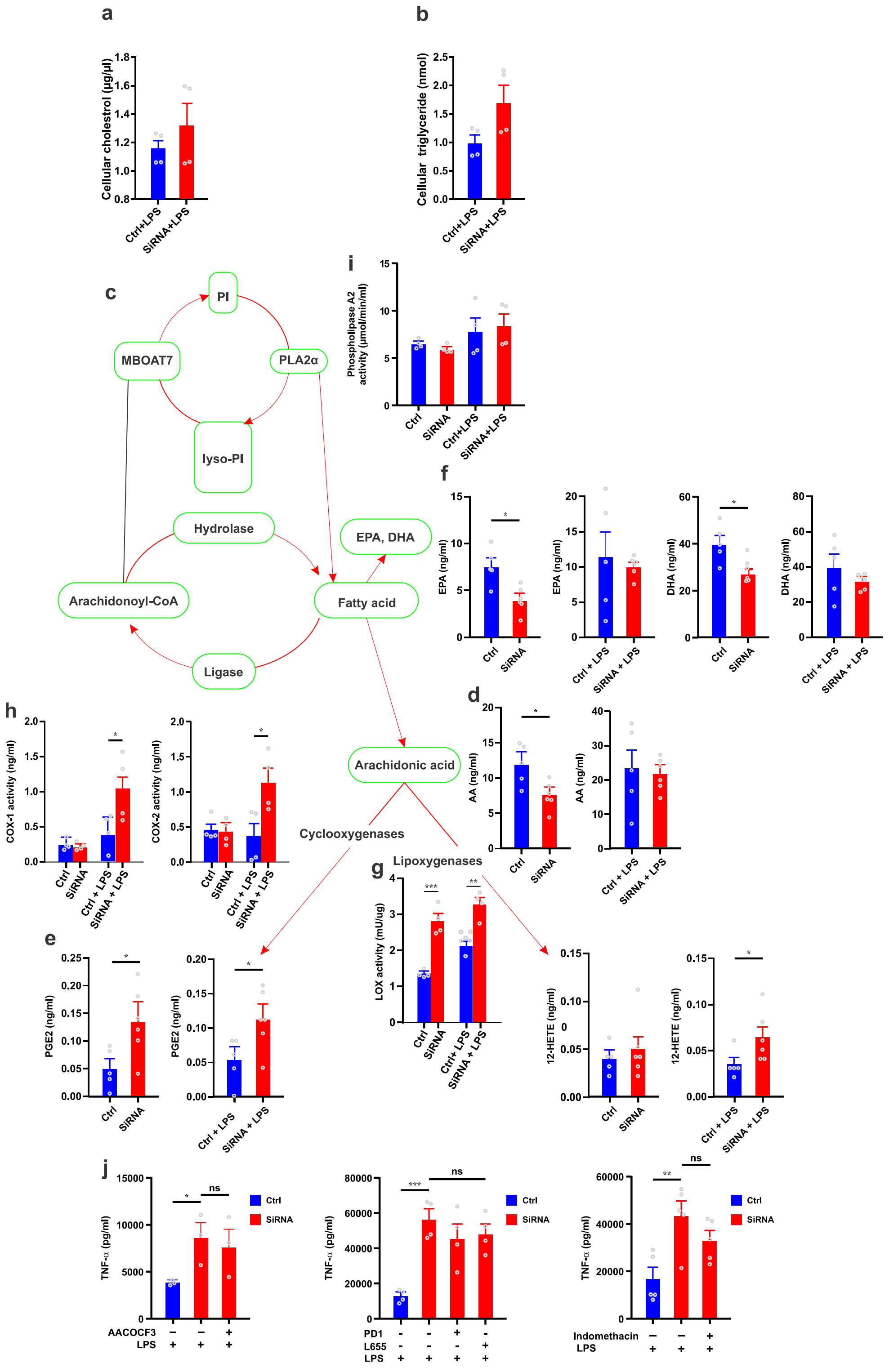
a
Cellular cholestrol (µg/µl)
Ctrl+LPS
SiRNA+LPS
b
Cellular triglyceride (nmol)
c
PI
MBOAT7
PLA2α
lyso-PI
Hydrolase
EPA, DHA
Arachidonoyl-CoA
Fatty acid
Ligase
Arachidonic acid
Cyclooxygenases
Lipoxygenases
i
Phospholipase A2 activity (µmol/min/ml)
f
EPA (ng/ml)
DHA (ng/ml)
d
AA (ng/ml)
h
COX-1 activity (ng/ml)
COX-2 activity (ng/ml)
e
PGE2 (ng/ml)
g
LOX activity (mU/ug)
12-HETE (ng/ml)
Ctrl
SiRNA
Ctrl + LPS
SiRNA + LPS
j
TNF-α (pg/ml)
AACOCF3
LPS
PD1
L655
Indomethacin
ns

Supplementary Figure 3: MDMs transfected with MBOAT7 siRNA or scramble at base line and challenged with LPS for 24 h and **a**) cholesterol and **b**) triglycerides were measured (n = 4 includes 2 biological replicates from independent individuals and 2 technical replicates). **c)** Scheme illustrating the role of MBOAT7 in the Land's cycle pathway. MDMs were transfected with MBOAT7 siRNA or scramble as control (n=6); **d)** levels of Arachidonic Acid, **e)** products of the cyclooxygenase (PGE2) and lipoxygenase pathway (12-HETE), **f)** pro-resolving n-3 fatty acids eicosapentaenoic acid (EPA) and docosahexaenoic acid (DHA), **g)** LOX enzyme activity (n=4), **h)** COX-I and COX-II enzyme activity (n=4), and **i)** Phospholipase A2 enzyme activity(n=4). **j**) Effect of inhibition of key enzymes in the eicosanoids production pathway on TNF-α secretion assessed by ELISA in conditional media of MDMs (n=3-5). P-value < 0.04 for all comparisons.Values are represented by vertical bars and are mean ± sem; *$P < 0.05$, ** $P < 0.01$, ****$P < 0.001$. Statistical differences between groups were assessed by 2-tailed Student's t test. Source data are provided as a Source Data file.

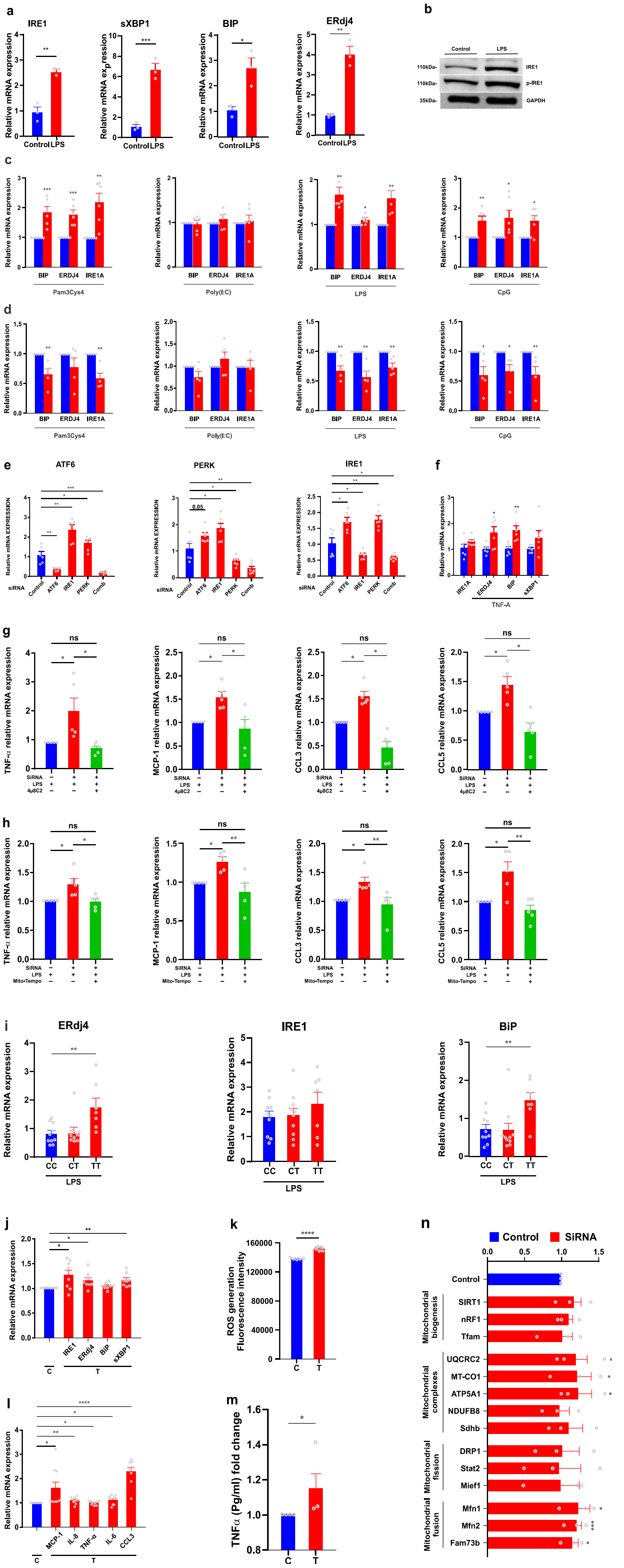
a
IRE1
sXBP1
BIP
ERdj4
Relative mRNA expression
Control LPS
b
Control
LPS
110kDa-
IRE1
110kDa-
p-IRE1
35kDa-
GAPDH
c
BIP
ERDJ4
IRE1A
Pam3Cys4
Poly(I:C)
LPS
CpG
d
e
ATF6
PERK
IRE1
Relative mRNA EXPRESSION
siRNA
Control
ATF6
IRE1
PERK
Comb
f
IRE1A
ERDJ4
BIP
sXBP1
TNF-A
g
TNF-α relative mRNA expression
MCP-1 relative mRNA expression
CCL3 relative mRNA expression
CCL5 relative mRNA expression
SiRNA
LPS
4μ8C2
ns
h
Mito-Tempo
i
ERdj4
IRE1
BiP
CC
CT
TT
LPS
j
IRE1
ERdj4
BIP
sXBP1
C
T
k
ROS generation
Fluorescence intensity
l
MCP-1
IL-8
TNF-α
IL-6
CCL3
m
TNFα (Pg/ml) fold change
n
Control
SiRNA
Mitochondrial biogenesis
SIRT1
nRF1
Tfam
Mitochondrial complexes
UQCRC2
MT-CO1
ATP5A1
NDUFB8
Sdhb
Mitochondrial fission
DRP1
Stat2
Mief1
Mitochondrial fusion
Mfn1
Mfn2
Fam73b

Supplementary Figure 4: MDMs were treated with LPS or control for 24h (n=3). Shown is **a)** Relative mRNA expression of different ER stress markers assessed by RT-PCR and **b)** IRE1 and p-IRE1 protein expression by Western blot. Relative mRNA expression of ER stress markers by RT-PCR in MDMs transfected with **c)** MBOAT7 siRNA or scramble or **d)** MBOAT7 overexpression plasmid or the empty vector after 24h of treatment with the indicated TLRs ligands (n = 5-6; 3 biological replicates and 1-2 technical replicates). **e)** Relative mRNA expression of ATF6, PERK and IRE1 by RT-PCR in MDMs transfected with scramble, ATF6 siRNA, IRE1 siRNA, PERK siRNA or combination of the three siRNAs for 48h and treated with LPS for 24h. **f)** Relative mRNA expression of different ER stress markers by RT-PCR in MDMs treated with TNF-α (n = 6; 3 biological replicates and 2 technical replicates). Relative mRNA expression of different cytokines by RT-PCR in MDMs transfected with scramble or MBOAT7 siRNA for 48h and treated with LPS only or in combination with **g)** 4μ8C (IREA1 inhibitor) or with **h)** Mito-TEMPO (mitochondria-targeted antioxidant) for 24h (n = 5; 3 biological replicates and 1-2 technical replicates). **i)** Relative mRNA expression of ER stress markers in MDMs from subjects of all genotypes for rs8736 and challenged with LPS for 24h (n=7–10/genotype). HeLa cells were transfected with full-length 3′UTRs of MBOAT7 containing either the C or the T allele (n=3) and analysed for; **j)** Relative mRNA expression of different ER stress markers, **k)** ROS generation, **l)** Relative mRNA expression of different cytokines (n = 8, 5, and 9; 3 biological replicates and 2-3 technical replicates, respectively) and **m)** Secreted TNF-α levels measured by ELISA (n=3). **n)** MDMs were transfected with scramble or MBOAT7 siRNA for 48h then examined for the relative mRNA expression levels of several regulators of mitochondrial dynamics(n=3). P-value < 0.04 for all comparisons. Values are represented by vertical bars and are mean ± sem; *P < 0.05, ** P < 0.01, ****P < 0.001. Statistical differences between groups were assessed by 2-tailed Student's t test or one-way ANOVA; multiple comparisons were corrected by Bonferroni correction. Source data are provided as a Source Data file.

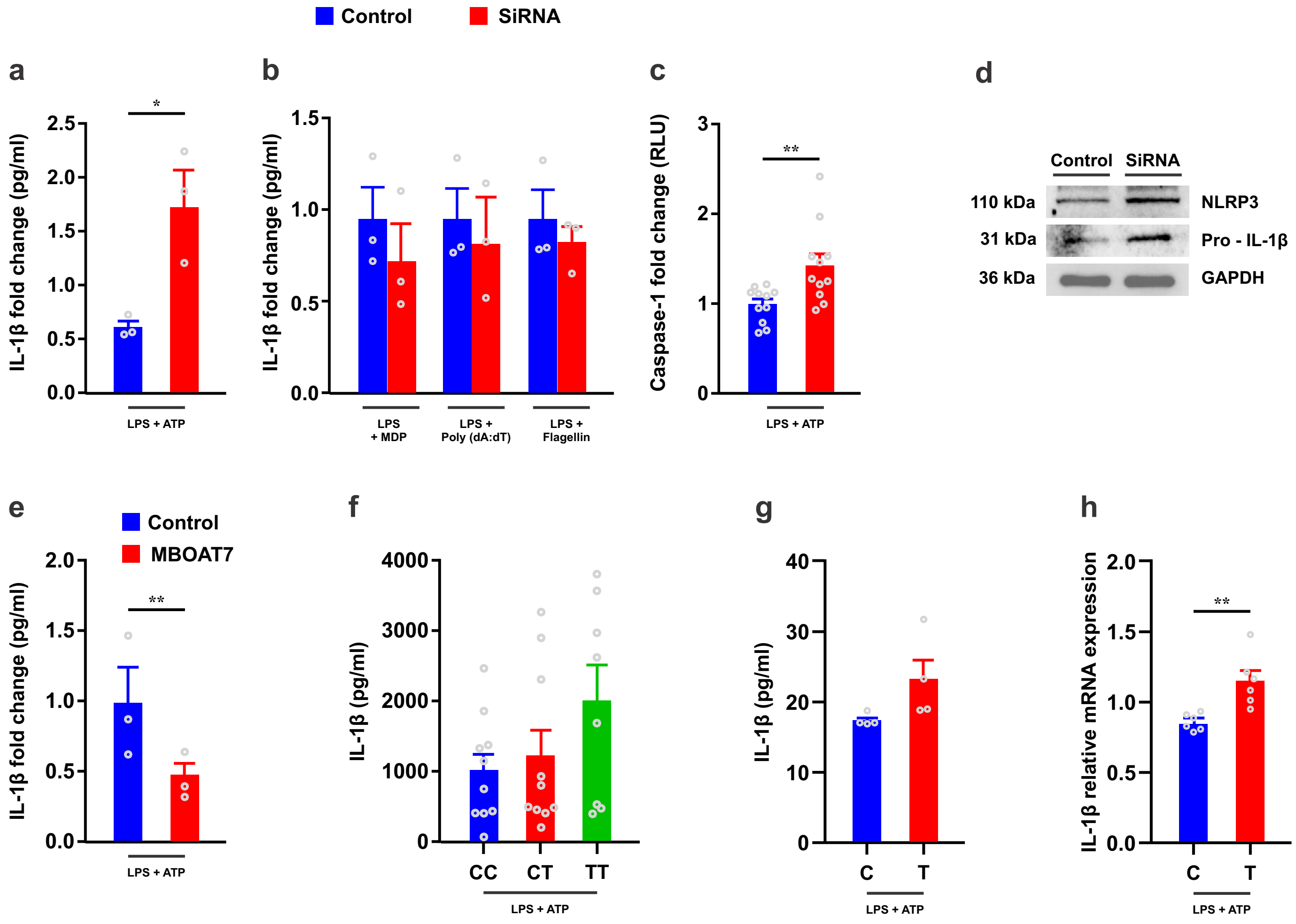

Control
SiRNA
a
b
c
d
IL-1β fold change (pg/ml)
*
2.5
2.0
1.5
1.0
0.5
0.0
LPS + ATP
1.5
1.0
0.5
0.0
LPS + MDP
LPS + Poly (dA:dT)
LPS + Flagellin
Caspase-1 fold change (RLU)
**
3
2
1
0
Control
SiRNA
110 kDa
NLRP3
31 kDa
Pro - IL-1β
36 kDa
GAPDH
e
f
g
h
Control
MBOAT7
IL-1β fold change (pg/ml)
**
IL-1β (pg/ml)
4000
3000
2000
1000
0
CC
CT
TT
LPS + ATP
40
30
20
10
C
T
IL-1β relative mRNA expression

Supplementary Figure 5: MDMs were transfected with MBOAT7 siRNA or scramble as control and incubated with LPS (500 ng/ml, 4 h) (n=3) and then **a**) treated with NLRP3 inflammasome activator ATP (5 mM, 30 min), **b**) NLRP1 inflammasome activator muramyl dipeptide (MDP) (200 ng/ml, 8h), the NLRC4 inflammasome activator flagellin ((200 ng/ml, 8h), or the AIM2 inflammasome activator poly(dA:dT) (1 ug/ml, 8h) and quantitative ELISA of the conditional media measuring IL-1β levels is shown. **c**) MBOAT7 silencing led to higher levels of active caspase-1(n = 11 includes 3 biological replicates from independent individuals and 3-4 technical replicates), **d**) and protein levels of pro- IL-1β and NLRP3 upon NLRP3 inflammasome activation **e**) MDMs were transfected with MBOAT7 overexpression plasmid or the empty vector as control and incubated with LPS (500 ng/ml, 4 h) (n=3) and then treated with NLRP3 inflammasome activator ATP (5 mM, 30 min) and quantitative ELISA of the conditional media measuring IL-1β levels is shown. **f**) MDMs (n = 10/genotype) were treated with LPS (500 ng/ml, 4 h) and then treated with NLRP3 inflammasome activator ATP (5 mM, 30 min) and the secreted IL-1β levels were measured by quantitative ELISA. Human HeLa cells were transfected with full-length 3′UTRs of MBOAT7 containing either the C or the T allele and incubated with LPS (500 ng/ml, 4 h) and then treated with NLRP3 inflammasome activator ATP (5 mM, 30 min) (n=4-6) analysed for **g**) IL-1β secretion using quantitative ELISA and **h**) IL-B mRNA expression using RT-PCR. Data was normalised to GAPDH. P-value < 0.02 for all comparisons.Values are represented by vertical bars and are mean ± sem; *P < 0.05, ** P < 0.01, ****P < 0.001. Statistical differences between groups were assessed by 2-tailed Student's t test or one-way ANOVA; multiple comparisons were corrected by Bonferroni correction. Source data are provided as a Source Data file.

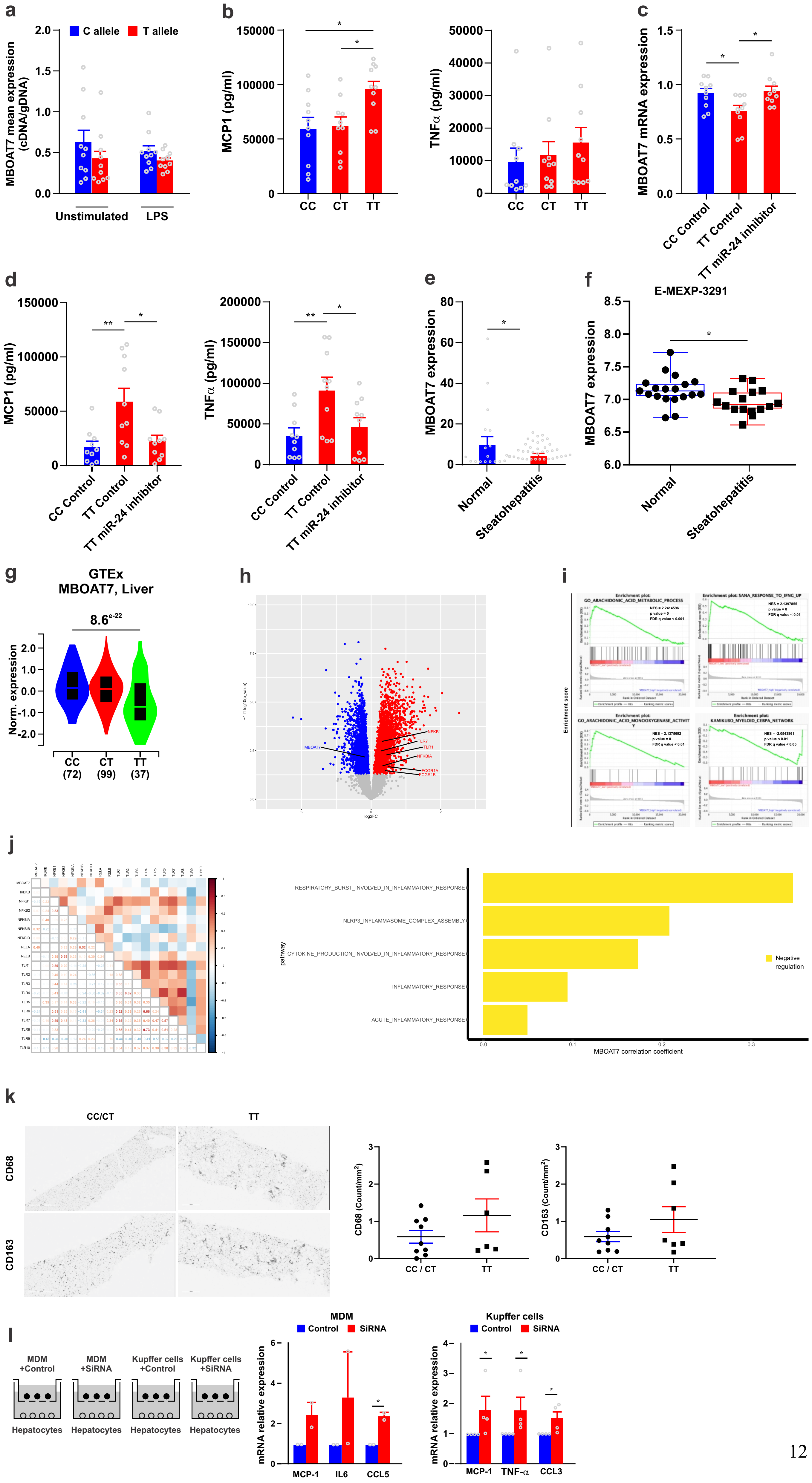

a
C allele
T allele
MBOAT7 mean expression (cDNA/gDNA)
Unstimulated
LPS
b
MCP1 (pg/ml)
TNFα (pg/ml)
CC
CT
TT
c
MBOAT7 mRNA expression
CC Control
TT Control
TT miR-24 inhibitor
d
MCP1 (pg/ml)
TNFα (pg/ml)
CC Control
TT Control
TT miR-24 inhibitor
e
MBOAT7 expression
Normal
Steatohepatitis
f
E-MEXP-3291
MBOAT7 expression
Normal
Steatohepatitis
g
GTEx
MBOAT7, Liver
8.6e-22
Norm. expression
CC (72)
CT (99)
TT (37)
h
i
j
RESPIRATORY_BURST_INVOLVED_IN_INFLAMMATORY_RESPONSE
NLRP3_INFLAMMASOME_COMPLEX_ASSEMBLY
CYTOKINE_PRODUCTION_INVOLVED_IN_INFLAMMATORY_RESPONSE
INFLAMMATORY_RESPONSE
ACUTE_INFLAMMATORY_RESPONSE
pathway
Negative regulation
MBOAT7 correlation coefficient
k
CC/CT
TT
CD68
CD163
CD68 (Count/mm²)
CD163 (Count/mm²)
CC / CT
TT
l
MDM +Control
MDM +SiRNA
Kupffer cells +Control
Kupffer cells +SiRNA
Hepatocytes
MDM
Kupffer cells
Control
SiRNA
mRNA relative expression
MCP-1
IL6
CCL5
TNF-α
CCL3

Supplementary Figure 6: Human MDMs isolated from patients with MAFLD and challenged with LPS for 24 hours (n= 10/group); **a)** Allele-specific expression assay showing the ratio of rs8736 alleles in genomic DNA (gDNA) and complementary DNA (cDNA) synthesized from pre-mRNA, **b)** Secreted MCP-1 and TNF-α levels measured by ELISA. MDMs from patients with MAFLD harbouring the CC genotype for rs8736 and transfected with miRNA control compared to subjects harbouring the TT genotype and transfected with either miRNA-24 inhibitor or control (n = 10/group); **c)** MBOAT7 relative mRNA expression assessed by RT-PCR , **d)** Secreted TNF-α and MCP-1 levels measured by ELISA. MBOAT7 relative mRNA expression **e)** by RT-PCR in control subjects (n=25) and patients with MAFLD related metabolic steatohepatitis (n=38), **f)** ArrayExpress public repository data (E-MEXP-3291) control subjects (n=19) and patients with MAFLD related metabolic steatohepatitis (n=16). **g)** MBOAT7 gene expression in the liver based on the Genotype-Tissue Expression (GTEx) dataset. **h)** Volcano plot and **i)** Gene Set Enrichment Analysis of inflammation pathways gene expression between the low and high expressed MBOAT7 samples in ArrayExpress public repository data (E-MEXP-3291). **j)** Heatmap of correlation (Pearson correlation) between hepatic MBOAT7 expression and inflammatory markers in E-MEXP-3291 dataset. Correlation between hepatic MBOAT7 expression and GSVA scores for inflammatory biological processes. **k)** Immunohistochemistry analysis of liver sections from patients with MAFLD harbouring CC/CT (n=9) or TT (n=6) genotype stained for sCD163 and CD68. Shown are representative immunohistochemistry images and summarized data. **l)** Diagram depicting the co-culture of primary human hepatocytes with MDMs or human Kupffer cells transfected with scramble or MBOAT7 siRNA for 48 hours and treated with LPS for 24 hours and relative mRNA expression of different cytokines in hepatocytes cultured with MDMs and with Kupffer cells assessed by q-PCR (n = 2 and 4 includes 2 biological replicates and 1-2 technical replicates respectively). P-value < 0.02 for all comparisons.Values are represented by vertical bars and are mean ± sem; *P < 0.05, ** P < 0.01, ****P < 0.001. Statistical differences between groups were assessed by 2-tailed Student's t test or one-way ANOVA; multiple comparisons were corrected by Bonferroni correction. Source data are provided as a Source Data file.

Supplementary 1 E:

Ctrl LPS

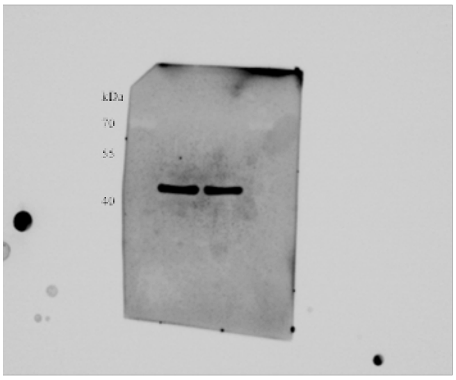

GAPDH

Ctrl LPS

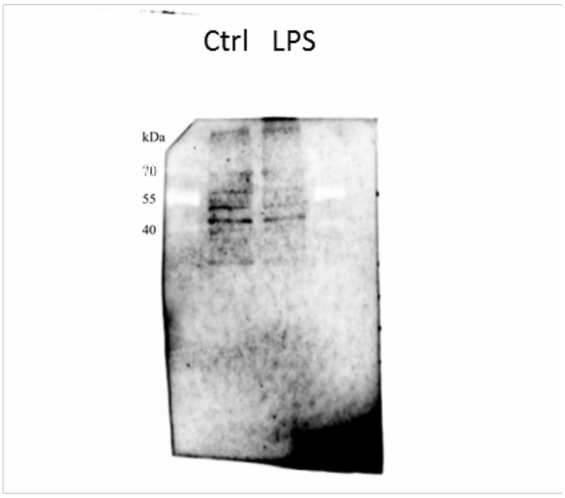

MBOAT7

Supplementary 2 A:

Control siRNA

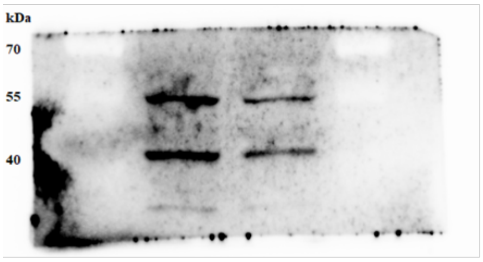

Vinculin

Control siRNA

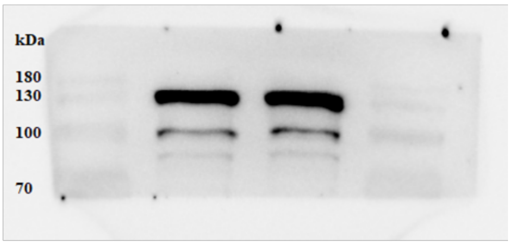

MBOAT7

Supplementary 4 B:

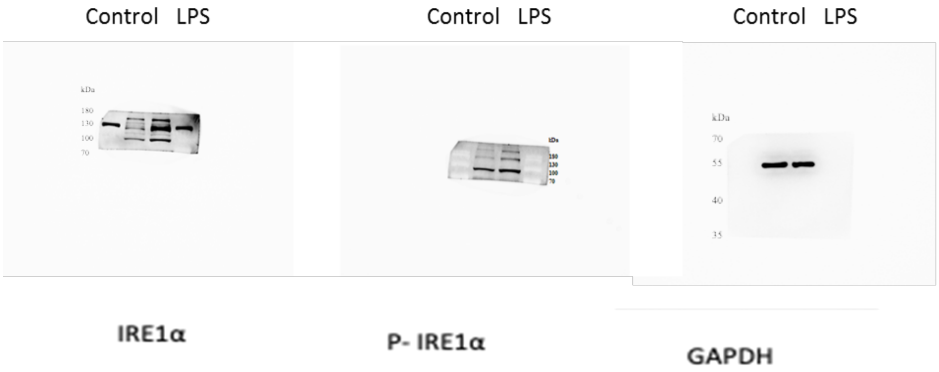

Supplementary 5 D:

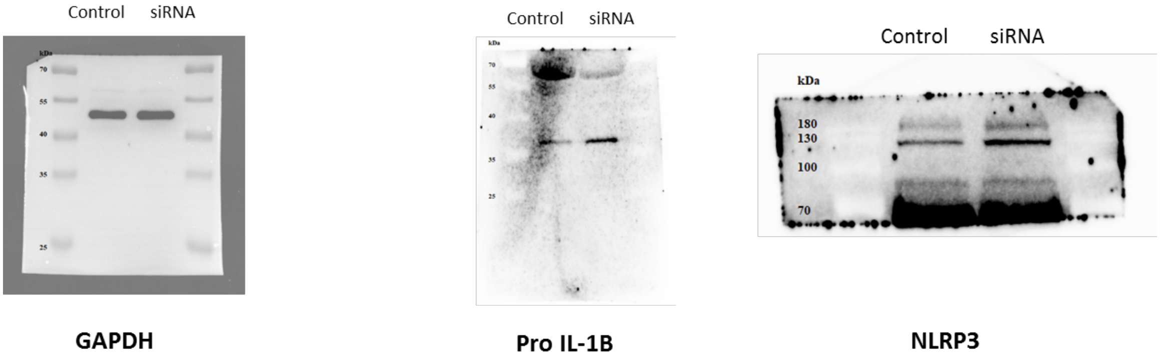

Figure 4A

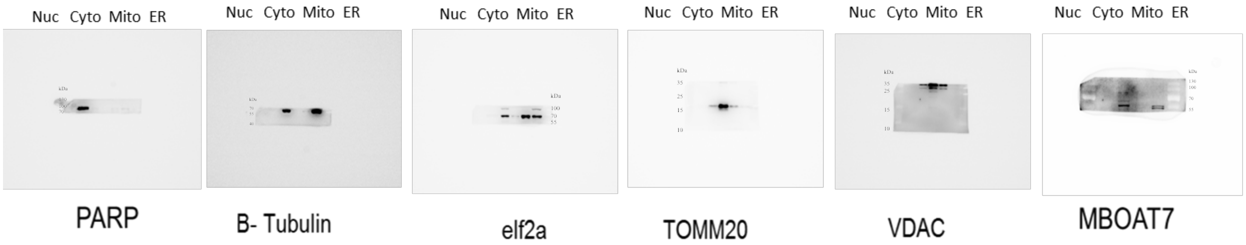

Figure 4B

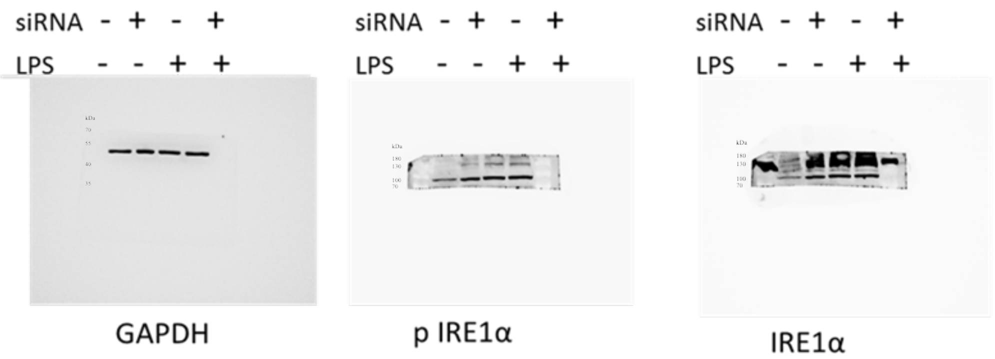

Figure 7H

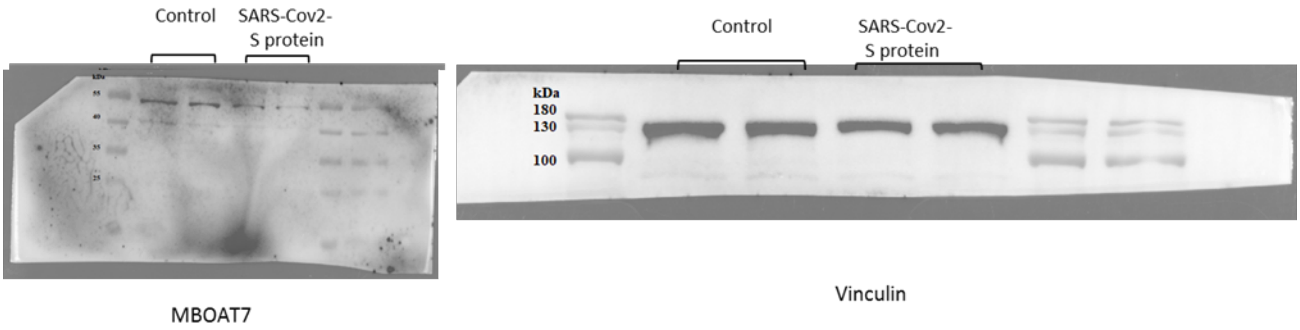

Figure 7 N

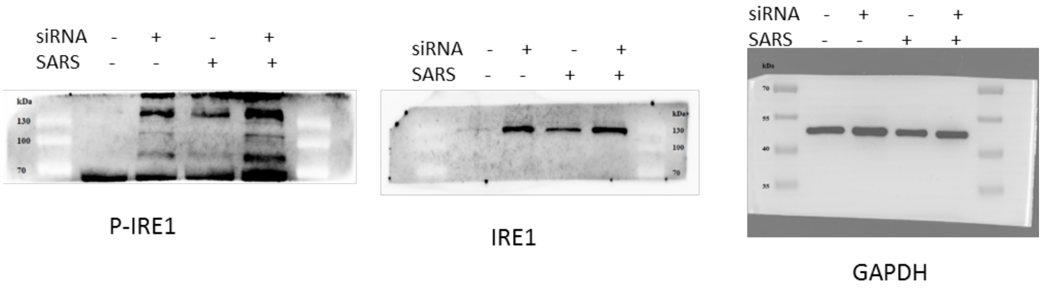
siRNA - + - +
SARS - - + +
kDa
130
100
70
P-IRE1
siRNA - + - +
SARS - - + +
kDa
130
100
70
IRE1
siRNA - + - +
SARS - - + +
kDa
70
55
40
35
GAPDH

Supplementary Table 1: Characteristics of the patient cohort with MAFLD

Variables	NAFLD Cohort (n=30)
**Age (years)**	58.5 (44.7-63)
**Male/ Female (%)**	17 (56.7%)/ 13 (43.3%)
**BMI (Kg/m^2)**	31.3 (25.5-36.5)
**Patients with diabetes' (%)**	17 (56.7)
**ALT (IU/L)**	51 (40.2-82.5)
**AST (IU/L)**	36 (19.7-58.5)
**GGT (IU/L)**	57.5 (30.7-177)
**Platelets ($x10^9/L$)**	214 (180-246)

*Data are given as median ± interquartile range or as %.

Supplementary Table 2: A list of the used reagents

**REAGENT or RESOURCE**	**Source / Identifier**
**Antibodies**	**Supplier name/ Catalog number / Clone name / Dilution**
anti-GAPDH	Abcam (ab181602), monoclonal, 1/10000
anti-Vinculin	Abcam (ab129002), monoclonal, 1/20000
anti-PARP	Cell Signaling Technology (9542), monoclonal, 1/1000
anti-β -Tublin	Cell Signaling Technology (2128), monoclonal, 1/1000
anti-EIF2A	Abcam (ab169528), monoclonal, 1/1000
anti-TOMM20	Abcam (ab186735), monoclonal, 1/1000
anti-VDAC	Abcam (ab14734), monoclonal, 1 μg/ml
anti-IL-1β	Cell Signaling (12242), monoclonal, 1/1000
anti-NLRP3	AdipoGen (AG-20B-0014), monoclonal, 1μg/ml
anti-pIRE1 alpha [p ser 724]	Novus Biologicals [NB100-2323], polyclonal, 1/1000
anti-IRE1 alpha	Novus Biologicals [NB100-2324], polyclonal, 1/1000
anti-MBOAT7	Sapphire Bioscience (ARP49810), polyclonal, 1/500
anti-CD163	Cell Marque, MRQ-26, monoclonal, 1/50
anti-CD68	Abcam, ab955, monoclonal, 1/500
**Bacterial and Virus Strains**	
NA	
**Chemicals, Peptides, and Recombinant Proteins**	
qScript cDNA SuperMix	Genescript
SYBR Green PCR Master Mix	Invitrogen
Bovine serum albumin (BSA)	Sigma Aldrich
RIPA buffer	Sigma Aldrich
Trypsin	Sigma Aldrich
Protease inhibitors	Sigma Aldrich
Dimethyl sulfoxide (DMSO)	Sigma Aldrich
β-mercaptoethanol,	Sigma Aldrich
EDTA (Ethylenediaminetetraacetic acid)	Sigma Aldrich
Actinomycin D	Sigma Aldrich
IL-β protein	Thermo fisher (A42508)

Penicillin	Gibco BRL through Life Technologies
Streptomycin	Gibco BRL through Life Technologies
Lipofectamine RNAi-MAX	Gibco BRL through Life Technologies
Transfection Reagent	Gibco BRL through Life Technologies
Opti-MEM medium	Gibco BRL through Life Technologies
Dulbecco’s Modified Eagle Medium (DMEM)	Lonza
RPMI 1640 Medium	Lonza
Phosphate buffered saline (PBS)	Lonza
Recombinant Human M-CSF	Lonza
TheraPEAKTM X-VIVOTM-15 Serum-free	Lonza
Hematopoietic Cell Medium,	Lonza
Ethanol, absolute	POCD Healthcare
Ficoll-Paque PLUS	GE Healthcare
Seahorse XF RPMI medium	In Vitro Technologies
Glutamine solution	In Vitro Technologies
Glucose solution	In Vitro Technologies
Pyruvate solution	In Vitro Technologies
Cryo Human Kupffer cells	Life Technologies
TaqMan™ Fast Advanced Master Mix	Thermo Fisher Scientific
**Critical Commercial Assays**	
Detergent Compatible (DC) Protein Assay Kit I	Bio-Rad (5000111)
FavorPrep Tissue Total RNA Purification Mini Kit	Favorgen Biotech Corp (FATRK001-2)
EasySep Human CD14 Positive Selection Kit II	STEMCELL Technologies (17858)
Human TLR1-9 Agonist Kit	InvivoGen (tlrl-kit1hw)
QIAGEN Plasmid Maxi Kit (25)	QIAGEN (12163)
QIAGEN miRNeasy Mini Kit	QIAGEN (217004)
DCFDA Cellular ROS Detection Assay Kit	Abcam (ab113851)
Human TNF-α ELISA Kit	Abcam (ab46087)
Human MCP-1 ELISA Kit	Abcam (ab179886)
Human IL-6 ELISA Kit	Abcam (ab178013)
Human IL-1β ELISA Kit	Abcam (ab214025)
Seahorse XF Cell Mito Stress Test Kit	In Vitro Technologies (SEA103015100)
Dual Luciferase (Firefly-Renilla) Assay System	JOMAR BIOSCIENCE (60683-2)

TMRE-Mitochondrial Membrane Potential Assay Kit	Abcam ab113852
MitoSOX™ Red Mitochondrial Superoxide Indicator	Thermo Fisher Scientific (M36008)
OneStep qMethyl-PCR Kit	ZYMO Research (D5310)
GSK1210151A	Sigma-Aldrich (SML0666-5MG)
Human Mitochondrial DNA Monitoring primer Set	Takara (7246)
Cytosolic Phospholipase A2 Assay Kit (ab133090)	Abcam (ab133090)
ATAC-Seq Kit	Active Motif (53150)
CUT&Tag-IT™ Assay Kit	Active Motif (53160)
Histone H3K27me3 antibody (pAb)	Active Motif (39055)
Histone H3K4me1 antibody (pAb)	Active Motif (39498)
ATP Colorimetric/Fluorometric Assay Kit	Biovision (K354-100)
Deproteinizing Sample Preparation Kit	Biovision (K808-200)
Lactate Colorimetric/Fluorometric Assay Kit	Biovision (K607-100)
Cyclooxygenase (COX) Activity Assay Kit (Fluorometric)	Abcam (ab204699)
Protease Inhibitor Cocktail (EDTA-free)	Abcam (ab201111)
Lipoxygenase Assay Kit	Abcam (ab241038)
Cytosolic Phospholipase A2 Assay Kit	Abcam (ab133090)
Fluo-8 Calcium Flux Assay Kit	Abcam (ab112129)
Caspase-1 Assay Kit	Promega Corporation (G9951)
Triglyceride Assay quantitation Kit	Abcam (ab65336)
Cholesterol/Cholesteryl Ester quantitation Kit	Abcam (ab65359)
Tocris AACOCF3	Invitro Tec. RDS (14625)
Indomethacin	Sigma-Aldrich (I7378-5G)
Actinomycin D	Sigma-Aldrich (A1410-2MG)
SARS-CoV-2 (2019-nCoV) Spike S1-His Recombiant Protein (HPLC-verified)	Jomar Life Research (40591-V08H-100UG)
Flagellin	Abcam (AB201366)
ATP	Sigma (A2383-1G)
Poly(dA:dT)	Jomar Life Research
MDP	Jomar Life Research
Mito-TEMPO	Sigma-Aldrich (SML0737)
Carbonyl cyanide 3-chlorophenylhydrazone (CCCP)	Sigma-Aldrich
Potassium chloride solution	Sigma-Aldrich

BAPTA-AM	Sigma-Aldrich
**Experimental Models: Organisms/Strains**	
NA	
**Oligonucleotides**	
Assay ID: Hs02786624_g1 / GAPDH	Thermo Fisher Scientific (4448489)
Assay ID: Hs04270764_pri, hsa-mir-23a	Thermo Fisher Scientific (4427012)
SNP ID: MBOAT7rs8736 C_2916339_10	Thermo Fisher Scientific (4351376)
mirVana™ miRNA Inhibitor, Negative Control	Thermo Fisher Scientific (4464076)
hsa-miR-24-2-5p mirVana® miRNA inhibitor	Thermo Fisher Scientific (4464084)
hsa-miR-24-2-5p mirVana® miRNA mimic	Thermo Fisher Scientific (4464066)
**Other**	
LENG4(MBOAT7) Human Tagged ORF Clone	OriGene
pCMV6-AC-GFP Tagged Cloning Vector	OriGene
siGENOME SMARTpool human MBOAT7	Dharmacon Cat. No. M-010084-01-0005
On-TARGET plus Non-Targeting pool	Dharmacon Cat. No. D-001810-10-05
LENG4 (MBOAT7) Human siRNA Oligo Duplex (Locus ID 79143)	ORIGENE Cat. No. SR312359
s18102 human PERK Silencer select	Thermo fisher (4427038)
s200430 human IRE1α Silencer select	Thermo fisher (4427038)
s223543 human ATF6 Silencer select	Thermo fisher (4427037)
TLR4 siRNA	Thermo fisher (4427038)
s13563 human STIM1 Silencer® Select	Thermo fisher (4427037)
s228396 human ORAI1 Silencer® Select	Thermo fisher (4427037)
Silencer™ Select Negative Control No. 1 siRNA	Thermo fisher 4390843
**Software and Algorithms**	
ImageJ bundled with 64-bit Java 8	https://wsr.imagej.net/distros/win/ij153-win-java8.zip
GraphPad Prism 9	http://www.graphpad.com/scientificsoftware/prism/
StepOne Software v2.3	https://www.thermofisher.com/hk/en/home/technical-resources/software-downloads/StepOne-and-StepOnePlus-Real-Time-PCR-System.html
R version 3.6.1	https://cran.r-project.org/bin/windows/base/old/3.6.1/
STAR version 2.7.2b (Dobin, 2013 #573)	https://www.ncbi.nlm.nih.gov/pubmed/23104886
Cuffdiff2 version 2.2.1 (Trapnell, 2013 #572)	https://bioinformaticshome.com/tools/rna-seq/descriptions/Cuffdiff-2.html

Ingenuity Pathway Analysis (IPA)	https://www.qiagen.com/us/products/discovery-and-translational-research/next-generation-sequencing/informatics-and-data/interpretation-content-databases/ingenuity-pathway-analysis/
Bowtie2 version 2.4.4	https://bowtie-bio.sourceforge.net/bowtie2/index.shtml
Homer version 4.11	http://homer.ucsd.edu/homer/
deepTools(version 3.5.1)	https://deeptools.readthedocs.io/en/develop/content/installation.html

Supplementary Table 3: A list of the sequence of primers

Primers	
GAPDH	For: AAGGTGAAGGTCGGAGTCAAG<br>Rev: GGGGTCATTGATGGCAACAATA
TNF-α	For: TCTCTAATCAGCCCT<br>Rev: TACAACATGGGCTAC
IL-6	For: TGCAATAACCACCCCTGACC<br>Rev: ATTTGCCGAAGAGCCCTCAG
MCP-1	For: TGC CGC CCT TCT GTG CCT G<br>Rev: ACA GCA GGT GAC TGG GGC AT
CCL3	For: TGCAACCAGTTCTCTGCATC<br>Rev: AATCTGCCGGGAGGTGTA
CCL5	For: CAACCCAGCAGTCGTCTTTG<br>Rev: TCCCGAACCCATTTCTTCTC
IL1RA	For: GTGCTTTGGTACAGGGATTCCTG<br>Rev: CACAGTCAGAGGTAGACCCTTC
MBOAT1	For: GTTTCCACAGCTTGCCAGA<br>Rev: AGGTGATGCCCAACTTGTGT
MBOAT2	For: TCTGGTGAAAATGGAAAAGAAGA Rev: GCTTCTGAACAACCGCAGTA
MBOAT3	For: TGACCTTTGATCGCTTCCAT<br>Rev: TCACCCTCGCGTCTCACT
MBOAT5	For: TGACATACCAGGAAAGATACCAAA Rev: GGTAGAAAAGGCCCAGACTCA
MBOAT7	For: CATGCGGTACTGGAACATGA<br>Rev: CCAGTAGGCGCTCAGCAG
BIP	For: TGTTCAACCAATTATCAGCAAACTC<br>Rev: TTCTGCTGTATCCTCTTCACCAGT
ERDJ4	For: GCTACTCCCCAGTCAATTTTCA<br>Rev: CCGATTTTGGCACACCTAAGAT
IREA1	For: GAAGATCCAGTCCTGCAGGTC<br>Rev: AGAAGAGAGGTTGATGGGCAG

sXBP-1	For: CTGCCAGAGATCGAAAGAAGGC<br>Rev: CTCCTGGTTCTCAACTACAAGGC
Affymetrix MBOAT7	For: AATATTGCAGACTTGGAAGG<br>Rev: CAACACTTTATTGGGAAAGATT
Zymo MBOAT7	For: TTCTACGTGGCCTGGATTG<br>Rev: GTTGCGGATGGTCTCATAGT
DRP1	For: CAAAGCAGTTTGCCTGTGGA<br>Rev: TCTTGGAGGACTATGGCAGC
STAT2	For: CCGGGACATTCAGCCCTTTT<br>Rev: CTCATGTTGCTGGCTCTCCA
MIEF1	For: AGGATGACAATGGCATTGGC<br>Rev: CCGATCGTACATCCGCTTAAC
PRKN	For: AACATCAGTAGCTTTGCACCTG<br>Rev: GGGGGAGTGATGCTATTTTT
NRF1	For: GGAGGAGTTCAATGAACTGCTGTC<br>Rev: CTCTGGACCTTCTGCTTCATCTGT
PGC-1α	For: AGCCTCTTTGCCCAGATCTT<br>Rev: GGCAATCCGTCTTCATCCAC
TFAM	For: GGCAAGTTGTCCAAAGAAACC<br>Rev: GCATCTGGGTTCTGAGCTTTA
UQCRC2	For: AATTTCGTCGTTGGGAAGTAGC<br>Rev: ATGAGTCTGCGGATTCTGAAAG
MT-CO1	For: GAGCTGCTGTTCGGTGTCC<br>Rev: TGCCAGTGGTAGAGATGGTTG
ATP5A1	For: ATGACGACTTATCCAAACAGGC<br>Rev: CGGGAGTGTAGGTAGAACACAT
SOD2	For: CTGCTGGGGATTGATGTGTGG<br>Rev: TGCAAGCCATGTATC TTTCAG T
MFN1	For: CCTGGCATCCAGGAGTTAGA<br>Rev: TGGTTCCAGCAATGCGATTT
MFN2	For: TGCAGGTGTAAGGGACGATT<br>Rev: GAGGCTCTGCAAATGGGATG
FAM73B	For: CTCCTGCAGGTGGTAGGC

	Rev: CAGAGACTGCATCAGAGCCA
SIRT1	For: TGGCAAAGGAGCAGATTAGTAGG<br>Rev: CTGCCACAAGAACTAGAGGATAAGA
PERK	For: GTCCCAAGGCTTTGGAATCTGTC<br>Rev: CCTACCAAGACAGGAGTTCTGG
ATF6	For: CAGACAGTACCAACGCTTATGCC<br>Rev: GCAGAACTCCAGGTGCTTGAAG
Catalase	For: AATCAGAAGGCAGTCCTCCC<br>Rev: TCGGGGAGCACAGAGTGTAC
NDUFB8	For: GCTCCCTGACCGCTCACAGC<br>Rev: TGCCAGTGCATCGGTTCACCC
SDHB	For: GACACCAACCTCAATAAGGTCTC<br>Rev: GGCTCAATGGATTTGTACTGTGC
p40phox	For: CCTATGACTCAGAGCAGGTG<br>Rev: TCTTCGTAGTAGTAGCAACG
p47phox	For: ACCCAGCCAGCACTATGTG<br>Rev: TTGCCTTCATCTGACAGAACC
p67-phox	For: CGAGGGAACCAGCTGATAGA<br>Rev: CATGGGAACACTGAGCTTCA
STIM1	For: 5- CACACTCTTTGGCACCTTCC-3'<br>Rev: 5-TGACAATCTGGAAGCCACAG-3'
ORAI1	For: 5-ACTGGATCGGCCAGAGTTAC-3'<br>Rev: 5-CGGCTCAAGTAGAGCTTGC-3'
IL-1β	For: 5’-CCACAGACCTTCCAGGAGAATG-3’<br>Rev: 5’-GTGCAGTTCAGTGATCGTACAGG-3’
