# Supplementary material for: A metabolic associated fatty liver disease risk variant in MBOAT7 regulates toll like receptor induced outcomes
Source: Nat Commun. 2022 Dec 6;13:7430. doi: 10.1038/s41467-022-35158-9 (PMC9726889; doi:10.1038/s41467-022-35158-9)
Supplement: Supplementary file 2 — Description of Additional Supplementary Files [file 41467_2022_35158_MOESM2_ESM.pdf]

Description of Additional Supplementary Files

File Name: Supplementary Data 1
Description: PheWAS of rs8736 with 1498 Phenotypes

File Name: Supplementary Data 2
Description: The impact of depletion of MBOAT7 on phospholipids in human macrophages. Multiple reaction monitoring (MRM) transitions used for detection of phospholipids.
