# Supplementary material for: A metabolic associated fatty liver disease risk variant in MBOAT7 regulates toll like receptor induced outcomes
Source: Nat Commun. 2022 Dec 6;13:7430. doi: 10.1038/s41467-022-35158-9 (PMC9726889; doi:10.1038/s41467-022-35158-9)
Supplement: Supplementary file 5 — Reporting Summary [file 41467_2022_35158_MOESM5_ESM.pdf]

nature portfolio

Corresponding author(s): Mohammed Eslam

Last updated by author(s): Sep 9, 2022

Reporting Summary

Nature Portfolio wishes to improve the reproducibility of the work that we publish. This form provides structure for consistency and transparency in reporting. For further information on Nature Portfolio policies, see our Editorial Policies and the Editorial Policy Checklist.

Statistics

For all statistical analyses, confirm that the following items are present in the figure legend, table legend, main text, or Methods section.

n/a	Confirmed	
☐	☒	The exact sample size (*n*) for each experimental group/condition, given as a discrete number and unit of measurement
☐	☒	A statement on whether measurements were taken from distinct samples or whether the same sample was measured repeatedly
☐	☒	The statistical test(s) used AND whether they are one- or two-sided<br>*Only common tests should be described solely by name; describe more complex techniques in the Methods section.*
☐	☒	A description of all covariates tested
☐	☒	A description of any assumptions or corrections, such as tests of normality and adjustment for multiple comparisons
☐	☒	A full description of the statistical parameters including central tendency (e.g. means) or other basic estimates (e.g. regression coefficient) AND variation (e.g. standard deviation) or associated estimates of uncertainty (e.g. confidence intervals)
☐	☒	For null hypothesis testing, the test statistic (e.g. *F*, *t*, *r*) with confidence intervals, effect sizes, degrees of freedom and *P* value noted<br>*Give P values as exact values whenever suitable.*
☒	☐	For Bayesian analysis, information on the choice of priors and Markov chain Monte Carlo settings
☒	☐	For hierarchical and complex designs, identification of the appropriate level for tests and full reporting of outcomes
☐	☒	Estimates of effect sizes (e.g. Cohen's *d*, Pearson's *r*), indicating how they were calculated

Our web collection on statistics for biologists contains articles on many of the points above.

Software and code

Policy information about availability of computer code

Data collection	No software was used for data collection.
Data analysis	The following softwares have been used to analyze data: R version 3.6.1, and GraphPad Prism version 9, STAR version 2.7.2b (Dobin, 2013 #573), Cuffdiff2 version 2.2.1 (Trapnell, 2013 #572), Ingenuity Pathway Analysis (IPA), Bowtie2 version 2.4.4, Homer version 4.11, deepTools(version 3.5.1), ImageJ (bundled with 64-bit Java 8).

For manuscripts utilizing custom algorithms or software that are central to the research but not yet described in published literature, software must be made available to editors and reviewers. We strongly encourage code deposition in a community repository (e.g. GitHub). See the Nature Portfolio guidelines for submitting code & software for further information.

Data

Policy information about availability of data

All manuscripts must include a data availability statement. This statement should provide the following information, where applicable:

- Accession codes, unique identifiers, or web links for publicly available datasets
- A description of any restrictions on data availability
- For clinical datasets or third party data, please ensure that the statement adheres to our policy

RNA-Seq, ATAC-seq and Cut&Tag datasets have been deposited at NCBI GEO dataset and are publicly available under the accession number: GSE20496 5. The rest

of the data are available within the main text and supplementary file. Source data are provided with this paper. The study did not generate new materials or codes.This paper analyses existing publicly available data. These datasets' accession numbers are (GSE158055, GSE164805, GSE171110, GSE173488, GSE160351, GSE157103 and E-MEXP-3291).

Human research participants

Policy information about studies involving human research participants and Sex and Gender in Research.

Reporting on sex and gender	Data are provided in supplementary table 1.
Population characteristics	The patients characteristics are provided in supplementary table 1. All volunteers were healthy on the day of sampling.
Recruitment	Patients consecutively recruited at Westmead Hospital, and Nepean hospital, Australia. Inclusion criteria were liver biopsy for suspected MAFLD with available DNA samples. Individuals with concomitant diagnoses were excluded, including excess alcohol intake (>20g per day for women and >30g per day for men), chronic viral hepatitis (hepatitis Band hepatitis C), autoimmune liver diseases, hereditary hemochromatosis, α1-antitrypsin, deficiency, Wilson's disease, and drug-induced liver injury.
Ethics oversight	Ethics approval was obtained from the Sydney West Area Health Service, the University of Sydney, Human Research Ethics Committee (HREC/17/WMEAD/433). Written informed consent was obtained from all participants.

Note that full information on the approval of the study protocol must also be provided in the manuscript.

Field-specific reporting

Please select the one below that is the best fit for your research. If you are not sure, read the appropriate sections before making your selection.

☒ Life sciences ☐ Behavioural & social sciences ☐ Ecological, evolutionary & environmental sciences

For a reference copy of the document with all sections, see nature.com/documents/nr-reporting-summary-flat.pdf

Life sciences study design

All studies must disclose on these points even when the disclosure is negative.

Sample size	No prior sample-size calculation was performed. Sample sizes were chosen based on standard requirements for biological analysis.
Data exclusions	No data was excluded in the study except one sample of eicosanoids assay due to technical issues.
Replication	There are at least two to three independent experiments. All replication attempts were successful. For the multi-omics analysis, all data are available to the public.
Randomization	For in vitro studies, primary cells have been allocated into the study groups based on the target genotype; immortalized cells have been allocated into the study groups randomly
Blinding	No blinded conditions used as blinding was not applicable to the study. Blinding was not used in this manuscript as it was not found relevant or possible for the applied experimental approaches

Reporting for specific materials, systems and methods

We require information from authors about some types of materials, experimental systems and methods used in many studies. Here, indicate whether each material, system or method listed is relevant to your study. If you are not sure if a list item applies to your research, read the appropriate section before selecting a response.

Materials & experimental systems

n/a	Involved in the study	
☐	☒	Antibodies
☐	☒	Eukaryotic cell lines
☒	☐	Palaeontology and archaeology
☒	☐	Animals and other organisms
☒	☐	Clinical data
☒	☐	Dual use research of concern

Methods

n/a	Involved in the study	
☒	☐	ChIP-seq
☒	☐	Flow cytometry
☒	☐	MRI-based neuroimaging

Antibodies

Antibodies used

anti-GAPDH (abcam, ab181602, monoclonal, 1/10000)
anti-Vinculin (abcam, ab129002, monoclonal, 1/20000)
anti-PARP (Cell Singnaling Technology, 9542, monoclonal, 1/1000)
anti-β -Tubulin (Cell Signaling Technology, 2128, monoclonal, 1/1000)
anti-EIF2A (Abcam, ab169528, monoclonal, 1/1000)
anti-TOMM20 (Abcam, ab186735, monoclonal, 1/1000)
anti-VDAC (Abcam, ab14734, monoclonal, 1 µg/ml)
anti-IL-1β (Cell Signaling, 12242, monoclonal, 1/1000)
anti-NLRP3 (AdipoGen, AG-20B-0014, monoclonal, 1µg/ml)
anti-pIRE1 alpha [p ser 724] (Novus Biologicals, NB100-2323, polyclonal, 1/1000)
anti-IRE1 alpha (Novus Biologicals, NB100-2324, Polyclonal, 1/1000)
anti-MBOAT7 (Sapphire Bioscience, ARP49810, Polyclonal, 1/500)
anti-CD163 (Cell Marque, MRQ-26, monoclonal, 1/50)
anti-CD68 (Abcam, ab955, monoclonal, 1/500)

Validation

The antibodies were validated according to the manufacturer.
The validation information of the primary antibodies used for immunobloting found on suppliers' webpages are as follow:

anti-GAPDH (suitable for Flow Cyt (Intra), WB, IHC-P, ICC/IF, IP; reacts with Mouse, Rat, Chicken, Human, Zebrafish, African green monkey, Xenopus tropicalis; https://www.abcam.com/gapdh-antibody-epr16891-loading-control-ab181602.html

anti-Vinculin (Suitable for: Flow Cyt (Intra), WB, IP, ICC/IF; reacts with: Mouse, Rat, Human; https://www.abcam.com/vinculin-antibody-epr8185-ab129002.html

anti-PARP (Suitable for: WB; reacts with: Human, Mouse, Rat, Monkey; https://www.cellsignal.com/products/primary-antibodies/parp-antibody/9542

anti-β -Tubulin (Suitable for: WB, IHC, IF, F; reacts with: Human, Mouse, Rat, Monkey, Zebrafish, Bovine; https://www.cellsignal.com/products/primary-antibodies/b-tubulin-9f3

anti-EIF2A (Suitable for: WB, IHC-P, ICC/IF; Knockout validated; reacts with: Mouse, Rat, Human; https://www.abcam.com/eif2a-antibody-epr11042-ab169528.html

anti-TOMM20 (Suitable for: Flow Cyt (Intra), ICC/IF, WB, IHC-P, IHC-Fr; reacts with: Mouse, Rat, Human; https://www.abcam.com/TOMM20-antibody-EPR15581-54-Mitochondrial-Marker-ab186735.html

anti-VDAC (Suitable for: WB, ICC/IF, Flow Cyt; reacts with: Mouse, Rat, Cow, Human; https://www.abcam.com/products/primary-antibodies/vdac1porin-antibody-20b12af2-ab14734.html

anti-IL-1β (Suitable for: WB, IHC; reacts with: Human, Mouse; https://www.cellsignal.com/products/primary-antibodies/il-1b-3a6-mouse-mab/12242

anti-NLRP3 (Suitable for: WB, ICC, IP, IHC, ChIP; reacts with: Human, Mouse; https://adipogen.com/ag-20b-0014-anti-nlrp3-nalp3-mab-cryo-2.html

anti-pIRE1 alpha [p ser 724] (Suitable for: WB, ChIP, ELISA, IB, ICC/IF, IHC, IHC-Fr, IHC-P, In vitro, IP, KD; reacts with: Human, Mouse, Rat, Porcine, Drosophilia, Goat, Mammal, Primate, Rabbit, Golden Syrian Hamster; https://www.novusbio.com/products/ire1-alpha-antibody_nb100-2323

anti-IRE1 alpha (Suitable for WB, ICC/IF, IHC, IHC-P, KD; reacts with: Human, Mouse, Rat; https://www.novusbio.com/products/ire1-alpha-antibody_nb100-2324

anti-MBOAT7 (Suitable for WB; reacts with: Human, Mouse, Rat, Cow, Dog, Guinea Pig, Horse; https://www.sapphirebioscience.com/product/NS00526944/leng4-peptide---c-terminal-region

anti-CD163 (suitable for IHC; reacts with paraffin; https://www.cellmarque.com/antibodies/CM/2010/CD163_MRQ-26

anti-CD68 (suitable for WB, ICC, IHC-P; reacts with Human; https://www.abcam.com/cd68-antibody-kp1-ab955.html

Eukaryotic cell lines

Policy information about cell lines and Sex and Gender in Research

Cell line source(s)

Primary human monocyte-derived macrophages were generated from healthy volunteers and patients with MAFLD. Hela

Cell line source(s)	cells, HEK293 and THP-1 cells were obtained from ATCC and Huh7 cells were obtained from cell bank Australia.
Authentication	Cell lines were authenticated via short-tandem repeat profiling.
Mycoplasma contamination	All cell lines were tested negative for Mycoplasma.
Commonly misidentified lines (See ICLAC register)	None.
